# Trehalose metabolism coordinates transcriptional regulatory control and metabolic requirements to trigger the onset of cassava storage root initiation

**DOI:** 10.1038/s41598-023-47095-8

**Published:** 2023-11-15

**Authors:** Nattavat Sukko, Saowalak Kalapanulak, Treenut Saithong

**Affiliations:** 1 Bioinformatics and Systems Biology Program, School of Bioresources and Technology and School of Information Technology, King Mongkut’s University of Technology Thonburi (Bang Khun Thian), Bangkok, 10150 Thailand; 2 School of Bioresources and Technology, King Mongkut’s University of Technology Thonburi (Bang Khun Thian), Bangkok, 10150 Thailand; 3 Center for Agricultural Systems Biology, Systems Biology and Bioinformatics Research Group, Pilot Plant Development and Training Institute, King Mongkut’s University of Technology Thonburi (Bang Khun Thian), Bangkok, 10150 Thailand

**Keywords:** Gene regulatory networks, Microarrays, Computational biology and bioinformatics, Regulatory networks, Systems analysis, Reverse engineering, Systems biology, Dynamic networks

## Abstract

Cassava storage roots (SR) are an important source of food energy and raw material for a wide range of applications. Understanding SR initiation and the associated regulation is critical to boosting tuber yield in cassava. Decades of transcriptome studies have identified key regulators relevant to SR formation, transcriptional regulation and sugar metabolism. However, there remain uncertainties over the roles of the regulators in modulating the onset of SR development owing to the limitation of the widely applied differential gene expression analysis. Here, we aimed to investigate the regulation underlying the transition from fibrous (FR) to SR based on Dynamic Network Biomarker (DNB) analysis. Gene expression analysis during cassava root initiation showed the transition period to SR happened in FR during 8 weeks after planting (FR8). Ninety-nine DNB genes associated with SR initiation and development were identified. Interestingly, the role of trehalose metabolism, especially trehalase1 (*TRE1*), in modulating metabolites abundance and coordinating regulatory signaling and carbon substrate availability via the connection of transcriptional regulation and sugar metabolism was highlighted. The results agree with the associated DNB characters of *TRE1* reported in other transcriptome studies of cassava SR initiation and *Attre1* loss of function in literature. The findings help fill the knowledge gap regarding the regulation underlying cassava SR initiation.

## Introduction

Cassava (*Manihot esculenta* Crantz) is a leading staple crop feeding almost one billion people annually^1^. Its underground roots, so-called starchy storage roots (SRs), comprise 70–90 percent of starch on a dry^2^. The yield of cassava is highly associated with the number of storage roots, root diameter, and root weight (volume)^3,4^. The number of storage roots is determined during tuber root initiation when fibrous roots (FR) develop into storage roots following bulking and starch accumulation^5,6^. Chiewchankaset et al. showed the high-yielding variety KU50 produced a significantly higher number of storage roots than the low-yielding variety HN at the root-bulking stage of plant development (~ 2–4 months after planting, MAP), and this difference was correlated with the final yield measured at ~ 10 MAP under same field conditions^7^. Similar observations are also reported in other independent studies with diverse cassava varieties and cultivation conditions^3,4,8^. These studies show that storage root initiation is equally important as the later developmental stage in achieving greater root yield. However, there is limited understanding of the processes in cassava, especially compared to sweet potato, another major tuber crop, and an improvement is vitally required.

Storage root initiation marks the transition from fiber-rich roots to starch-rich bulky roots, characterized by the thickening of fibrous roots from secondary cell growth driven by vascular cambium and starch accumulation in the vascular parenchymatous cells^5,9–11^. The thickening roots can be divided into three developmental phases simply by root diameter: fibrous roots (FR) < 2 mm in diameter, intermediate roots (IR) ~ 2–5 mm in diameter, and storage roots (SR) > 5 mm in diameter^6^. The transition from fibrous to storage roots is tightly regulated by transcriptional regulation and hormones^12–16^. Secondary growth of vascular cells and cell division during root development in Arabidopsis are modulated by the action of transcription factors (TFs), as revealed by genome-wide gene expression^16^. In rice, the interaction of TFs and phytohormones is required to induce root meristem activity and promote root growth^17^. Similar regulation is also found in tuber crops such as sweet potato and cassava, where the synchronous harmony of transcriptional regulation and phytohormones determines the fate of storage root initiation^10,12–15,18–20^. Transcriptome analysis of early developing storage roots showed that *KNOX1*, a TF related to root development^21,22^, played a key role in combining the action of multiple phytohormones and TFs to reach the onset of cassava SR formation^14^. Gene regulation and phytohormone signaling have provided insights into the complex regulation of cassava storage root initiation and development, but there is still limited understanding of the crucial metabolism for providing sufficient substrates for root bulking. The recent discovery of *lbNAC083*’s role in regulating pencil root transition in sweet potato by controlling lignification and starch biosynthesis suggests a strong link between metabolism and transcriptional regulation^20^.

During the transition to storage roots, plants require enormous carbon substrates as well as energy^23,24^. Metabolism, especially carbon assimilation, is thus one of the crucial factors determining the onset of storage root initiation^24^. Sucrose is produced as a primary carbon substrate in source tissues by photosynthesis and is subsequently allocated to root sink tissues for biomass synthesis and starch production^24,25^. In radish, sucrose metabolism is activated during storage root development, making it essential for root growth and development^25^. Similarly, genes and metabolic pathways involved in sucrose metabolism are highly active during tuberization in sweet potato and cassava^26,27^. In addition to supplying carbon substrates used as the building block for anabolic processes, sucrose functions as a signaling molecule in a wide range of cellular activities. For instance, it delivers the signal to trigger root meristem development in Arabidopsis by activating the protein kinase associated with central growth regulators^28,29^. Sucrose is also reported to induce starch biosynthesis metabolism in leaves of Arabidopsis and sweet potato by inducing the expression of *AtWRKY20*, a transcriptional activator of *APL3* (ADP-glucose pyrophosphorylase large subunit 3) and *APS1* (ADP-glucose pyrophosphorylase small subunit 1) genes encoding large and small subunits of AGPase (ADP-glucose pyrophosphorylase) enzyme, respectively^30^. Trehalose-based sugars are another well-known signaling molecule that coordinates a spectrum of regulatory pathways^31^. Particularly, trehalose-6-phosphates (T6P), an intermediate in trehalose metabolism, conveys a negative feedback signal to re-adjust the optimal level of sucrose for signifying the stage of development between vegetative shoot growth and root sink organs formation^31,32^. The involvement of T6P in the regulatory cascade of root development has been reported, including comprehensive reviews^33,34^. T6P inhibits SnRK1 (Sucrose non-Fermenting Related Kinase 1), key mediating regulators linking sugar metabolism to plant development, and modulates the action of TOR (Target of Rapamycin) in the central growth regulatory pathway of plant development^35^. Perturbation of T6P metabolism affects the expression of SnRK1 and related genes involved in cell proliferation, resulting in delayed sprouting time, tuber carbohydrates and tuber yield^36^. T6P influences starch synthesis by regulating AGPase enzyme activity at redox reaction^37,38^. The role of T6P in sugar-based signaling is often overlooked in transcriptome analysis due to the unclear expression footprint of the related genes by nature of the fine regulatory control process.

The transition from fibrous to storage roots which driven by a dynamic regulatory process, of which the associated regulators are hard to capture through the typical method of differential gene expression (DEG) analysis. Weighted Gene Correlation Network Analysis (WGCNA) was introduced as an effective approach to identify regulators and their association to stages/traits based on the similarity of gene expression patterns across samples^39,40^. The method is successful to unravel complex biological regulation in plants^41^. However, the given results are always penalized by the strictly significant analysis and exclude the highly variated samples, which is one of the notified characters of genes involving in transition process^42,43^. The recently proposed Dynamic Network Biomarker (DNB) offers a promising solution to address the challenge of capturing the dynamic regulatory events that occur during the transition process. DNB is a model-free approach to seek key regulators involved in the biological phase transition^43,44^. The method borrows the bifurcation theory to define gene expression characteristics of regulators that move forward the critical stage (tipping point) of the system, then entering the new state. DNB genes are basically characterized based on a strong correlation within the gene set and a weak association with other outside groups, resulting in hyper-variant expression among samples at the pre-transition state. The DNB method has been used to explore the critical state of disease progression and identify regulators driving state transition^43,45–52^. This conceptual method has also been proven well applicable to study transient regulation in plant development and diseases. It was able to identify genes modulating flowering transition in Arabidopsis^53^, pencil root formation in sweet potato^20^, fruit ripening transition in grapevine^54^, MeJA-induced growth-to-defense transition in plants^55^, tobacco etch disease response^56^ and drought stress response in Arabidopsis^57^.

In this study, we aimed to investigate the regulation underlying cassava storage root initiation based on DNB analysis of the entire gene expression in developing roots. The gene expression profile changed substantially and globally from fibrous to storage roots, demonstrating the occurrence of state transition. A group of 99 DNB genes identified herein was proposed to play a role in the transition into the storage root formation stage. Some of the resulting DNBs had previously been linked to storage root development, for example, ethylene-responsive transcriptional factors (*ERF*), sterol methyltransferase 1 (*SMT1*), anthranilate synthase 2 (*ASA2*), and sulfotransferase 17 (*SOT17*). Besides, our analysis showed that trehalase1 (*TRE1*), an enzyme modulating T6P abundance in trehalose metabolism, potentially played a crucial role in connecting transcriptional regulation to sugar metabolism and determined the onset of SR initiation. The results are in agreement with the associated expression of *TRE1*, as markedly shown in various transcriptome studies of SRs initiation, and the observed levels of key sugars, including sucrose and T6P, patternized during developmental state transition. The insights gained here help narrow the knowledge gap on regulatory mechanisms underlying cassava SR initiation, especially with regard to the connection between the transcriptional regulatory cascade and metabolic control. Our findings shed light on the controllability of SR initiation, which is one promising strategy for boosting cassava SR yields.

## Results

### Global and substantial changes in gene expression demonstrated the transition from fibrous to storage roots

**Figure 1:**
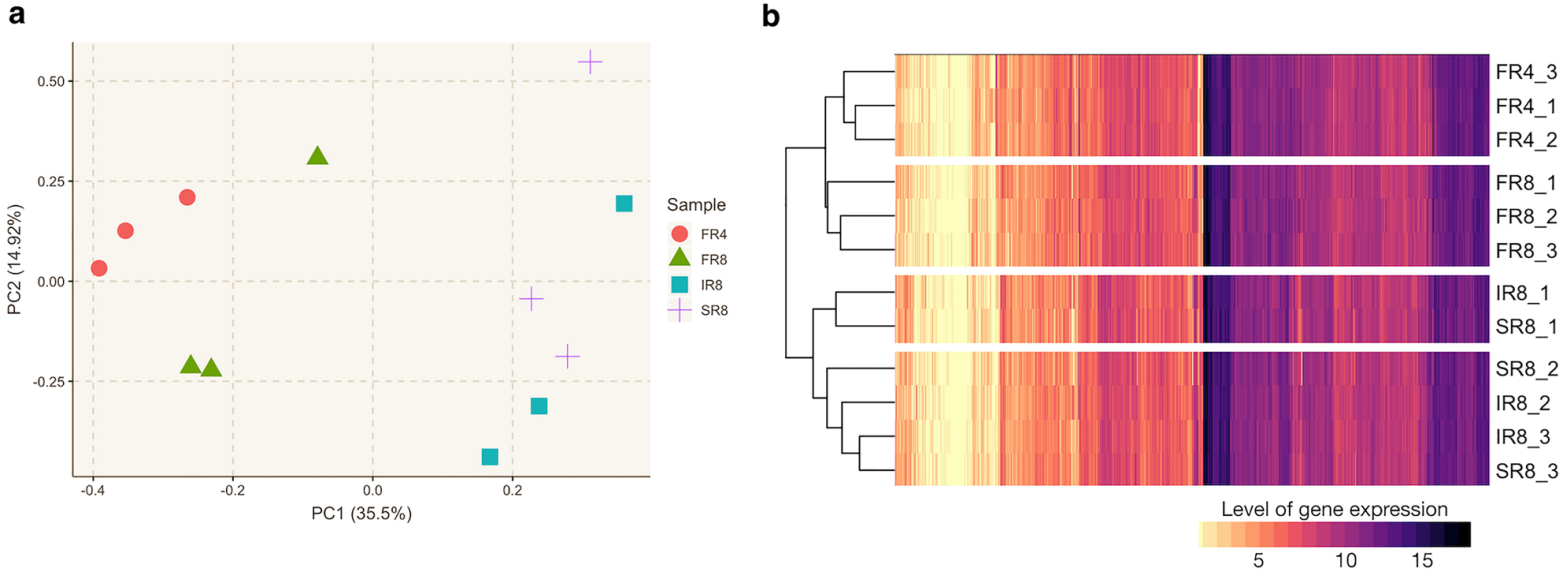
Overall gene expression profiles in developing roots of cassava. (**a**) Principal Component Analysis (PCA) of genome-wide gene expression in triplicate samples of fibrous (FR), intermediate (IR) and storage (SR) roots of four- and eight-weeks-old plants, denoted as FR4, FR8, IR8, and SR8. (**b**) Unsupervised hierarchical clustering of gene expression in corresponding root samples. Colors in the heatmap represent levels of gene expression from low (yellow) to high (black).

Transcriptome data of developing storage roots of cassava acquired from 8-week-old plants^14^ was employed to study the regulation underlying the onset of SR formation. Genome-wide gene expression in fibrous [FR, at four (FR4) and eight (FR8) weeks], intermediate [IR, at eight weeks (IR8)], and early developing storage roots [SR, at eight weeks (SR8)] was measured using a 60-mer-oligo microarray. Overall gene expression in all root types was analyzed and compared, as shown in Fig. 1. Principal component analysis (PCA) showed differences in gene expression profiles by age and developmental stages of roots (Fig. 1a). Overall gene expression of FR4 was distinct from that of FR8, and both differed from IR8 and SR8, which showed more similar profiles. Correspondingly, unsupervised hierarchical clustering analysis classified the expression of FR4 and FR8 into two separate groups but could not clearly distinguish between the gene expression of IR8 and SR8 (Fig. 1b). Global gene expression analysis revealed changes in transcriptional activity associated with the stages of root development, from fibrous roots to storage roots. The more similar overall gene expression of IR8 to SR8, in comparison to FR8, suggested that IR8 was at the early stage of SR development. In addition, it was observed that gene expression profiles varied largely among replicates in roots of 8 weeks-old plants. The replicate-level variations in the gene expression profiles, determined by the coefficient of variance (CV) (Supplementary Fig. S1), were observed at eight weeks, suggesting the transition period of SR development. All root types (FR, IR, and SR) showed significantly higher CV at 8 weeks than FR at four weeks, indicating the stage transition.

### Analysis of dynamic changes in gene expression, and identification of key regulatory genes modulating storage root initiation

**Figure 2:**
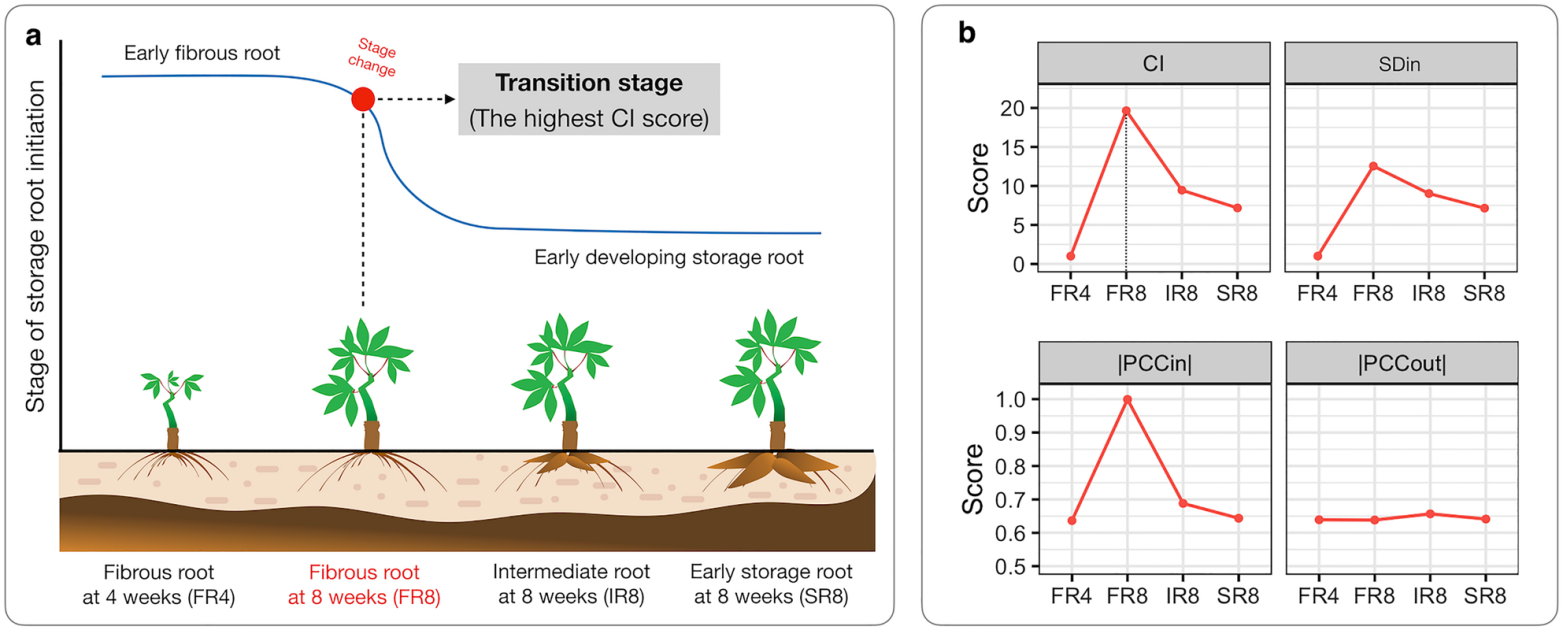
DNB analysis of gene expression in developing cassava roots. (**a**) Schematics of stage transition from fibrous to storage roots at four (FR4) and eight weeks (FR8) of growth. FR8 was considered the transition stage (critical stage) based on the highest composite index (*CI*) score, which combines the following criteria (**b**): (i) highly variable expression among samples ($\it \it {\text{SD}}_{\text{in}}$), (ii) high within-group association of DNB genes ($\it \it \left|{\text{PCC}}_{\text{in}}\right|$), and (iii) weak correlation with other genes (or non-DNB genes) ($|{PCC}_{out}|$)^43^.

**Figure 3:**
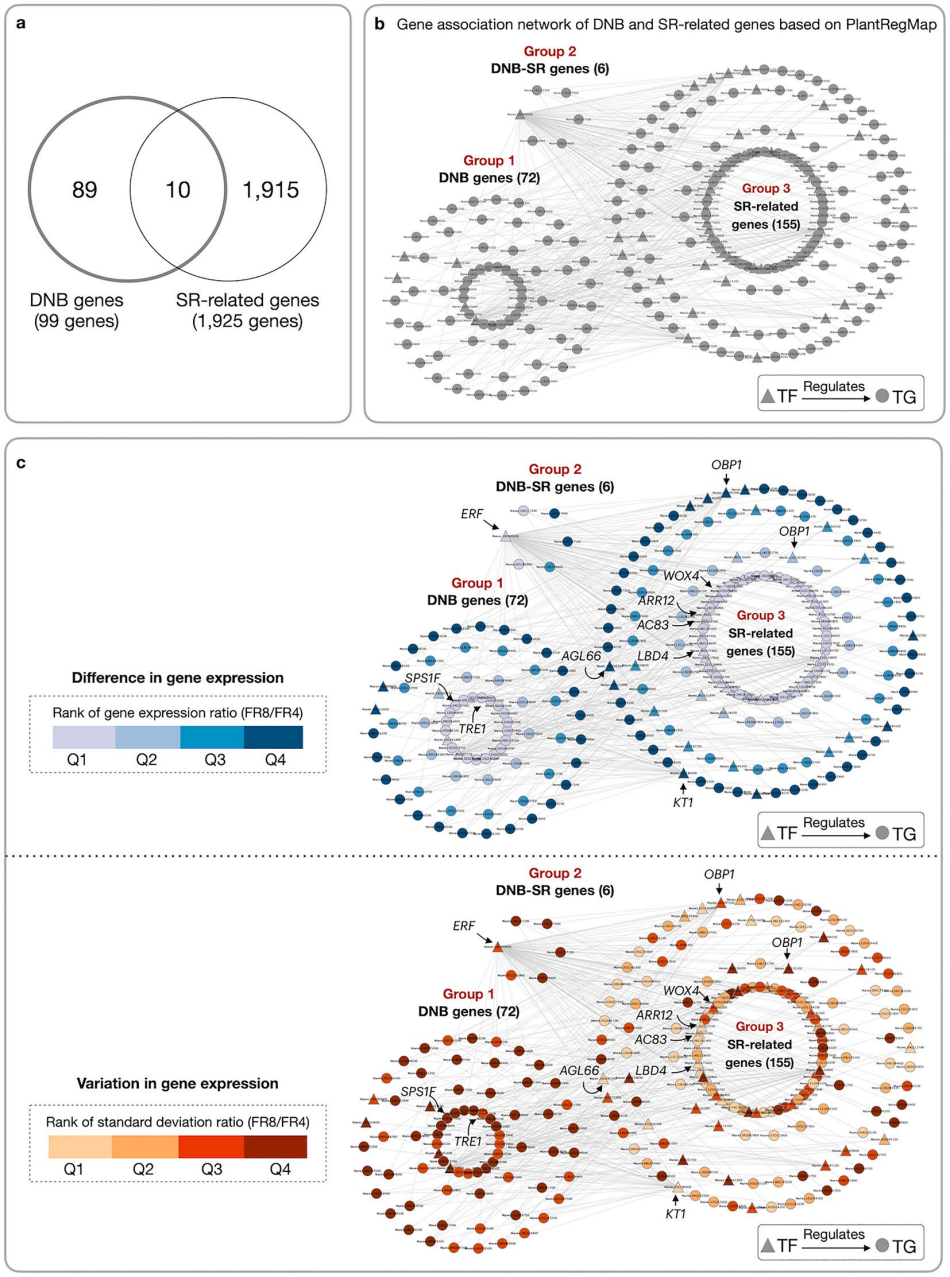
Comparison of DNB and SR-related DEG genes. (**a**) Venn diagram of DNB and DEG genes from cassava literature^10,13–15^. (**b**) Transcriptional regulatory network (TRN) of DNB and DEG genes constructed based on cis-regulatory elements of cassava in the PlantRegMap database^58^. The network is divided into three groups: (1) DNB genes (72 genes), (2) SR-related DEG genes (155 genes) and (3) overlapping DNB and DEG genes (6 genes). Triangular and circular nodes denote transcription factors (TFs) and target genes (TGs), respectively. Arrows represent TFs and TGs association inferred from the PlantRegMap database. (**c**) Gene expression levels (top) and variance (bottom) of DNB and DEG genes in FR8. All gene expression data were analyzed relative to FR4’s.

Gene expression changed dynamically at the onset of SR development. DNB analysis identified the transition point (tipping point) based on the magnitude of the composite index (*CI*) score, which is a combined signal indicator of potential key regulators, namely DNB genes. DNB genes were identified at each stage according to high variability in expression profiles of the replicates and the influence of such changes on gene relationships in the transcriptional regulatory cascade. Three criteria determine the critical point of a system: (i) high variable expression among samples ($\it \it {\text{SD}}_{\text{in}}$), (ii) high correlation within a group of DNB genes ($\it \it \left|{\text{PCC}}_{\text{in}}\right|$), and (iii) weak correlation with other genes (or non-DNB genes) ($|{PCC}_{out}|$)^43^. Our analysis showed a peak *CI* score at FR8, suggesting a transition point for SR initiation (Fig. 2). A total of 99 DNB genes were identified (Fig. 3, Supplementary Data S1), of which 10 SR-related genes obtained by differentially expressed gene analysis (DEG-based approach) had been reported in cassava literature, including the ethylene-responsive transcription factor (*ERF*), which plays an essential role in modulating cassava root bulking by attenuating the gibberellic acid level^14^, sterol methyltransferase 1 (*SMT1*), which is involved in the production of brassinosteroid hormone essential for cassava root development^15^, and sulfotransferase 17 (*SOT17*), involved in controlling vascular cambium activity required for secondary cell growth during SR formation^10^. These 99 DNB genes, in addition, included 7 transcription factors (TFs) genes, 5 phytohormone-related genes, 4 kinase genes, 4 genes involved in secondary cell growth, and 7 genes involved in carbohydrate metabolism (see also Supplementary Data S1 for an entire list). The description of the 99 genes’ functions demonstrated their associative role at the early stage of SR formation and probably also at the later stage of SR development.

The majority of the DNB genes (89 of 99 genes) identified here are exclusive to the DNB approach, i.e., they are not part of the previously reported DEG-derived 1,925 SR-related genes (Supplementary Data S2) (Fig. 3a). The relationship between the two gene sets (DNB and DEG) was investigated through a transcriptional regulatory network (TRN) developed based on the PlantRegMap database. Figure 3b shows the TRN network of transcription factors (TF) and their target genes (TG) for the 99 DNB and 1925 SR-related genes. The network contains, in total, 233 genes, 46 TFs, 187 TGs and 343 interactions. The 233 genes comprise 72 DNB genes (Fig. 3b, Group 1), 155 DEG genes (Fig. 3b, Group 3), and 6 genes found in both groups (Fig. 3b, Group 2). Although the two gene sets were minimally overlapped, they were highly connected, indicating a difference in sub-processes associated with SR development.

The DNB and DEG genes were further evaluated for differences in variation and gene expression relative to control conditions (FR4). Figure 3c (top panel) showed the majority of genes in both sets were expressed differentially to FR4. The DEG genes were more steadily expressed with less variance among replicates, including the KNOTTED-like (*KT1*) gene, NAC domain-containing protein 83 (*AC83*), AGAMOUS-like 66 (*AGL66*) and response regulator 12 (*ARR12*)—which are related to phytohormones, and LOB domain-containing protein 4 (*LBD4*)—involved in secondary cell growth regulation^10,13^. By contrast, the DNB genes, including carbon metabolic genes such as sucrose phosphate synthase 1F (*SPS1F*) and trehalase 1 (*TRE1*) involved in starch metabolism^59^, showed highly variable expression among samples (Fig. 3c, bottom panel), reflecting the tipping point of state transition. Overall, the DNB genes, especially the 89 genes exclusively identified here, appeared more relevant to the regulation of SR initiation and FR-to-SR transition, while the DEG genes were mainly active at the later SR maturation stage.

### Trehalose metabolism might play a dominant role in regulating storage root initiation

**Figure 4:**
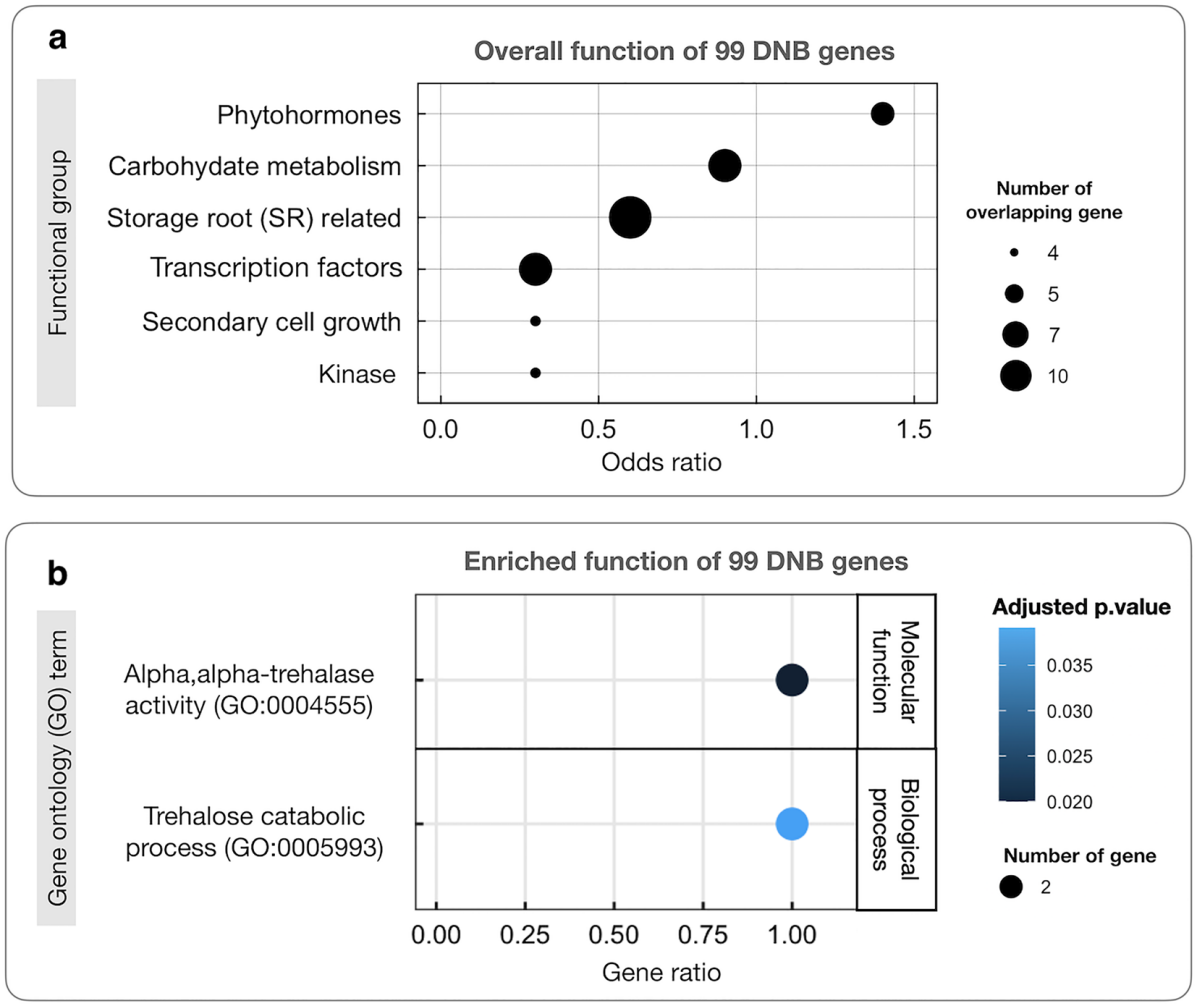
Functional analysis of 99 DNB genes. (**a**) The association of DNB with six functional gene groups relevant to storage root initiation, obtained from databases and cassava literature. Odds ratio represents the association between DNB and functional genes. Circle size in 4a denotes the number of DNB genes overlapped with functional genes. (**b**) GO enrichment analysis of 99 DNB genes. Node colors represent the significance levels of the enriched terms according to the adjusted *p*-value (FDR; Benjamini-Hochberg). Gene ratio is the proportion of DNB genes and total genes in the genome annotated to the GO term. Circle size in 4b represents the number of DNB genes in the GO term.

Functional analysis of the 99 DNB genes was first done with gene groups that are highly relevant to storage root development, including phytohormones, carbohydrate metabolism, TFs, secondary cell growth, kinase-related genes, and SR-related genes identified in cassava literature. Results showed the DNB genes were functionally associated with phytohormones (odds ratio ≥ 1) and carbohydrate metabolism (odds ratio approximately ~ 1) (Fig. 4a). Five DNB genes were related to phytohormones, namely cullin1 (*CUL1*), lipoxygenase 1 (*LOX1*), sterol methyltransferase 1 (*SMT1*), anthranilate synthase 2 (*ASA2*) and ATP binding cassette subfamily B19 (*ABCB19*). These genes are also linked to SR formation. Mutation of gene *CUL1* in Arabidopsis reduced the expression of auxin reporters, resulting in a low response to auxin signaling and fewer lateral roots formation compared to the wild-type^60^. Expression of *LOX* genes are influenced by various plant hormones, including auxin, gibberellin, kinetin (cytokinin) and salicylic acid^61^. The loss of *LOX1* function alters potato tuberization, reducing the tuber size and final yield^62^. *SMT1* is involved in brassinosteroid biosynthesis, required to induce tuber root formation in cassava^15^. *ASA2* encoding anthranilate synthase is essential for producing tryptophan, a primary precursor in the auxin biosynthesis pathway^63^. Alteration of *ABCB19* auxin transporter gene expression in Arabidopsis varies auxin transportation, affecting both adventitious^64^ and lateral root^65^ formation. Seven DNB genes were involved in carbohydrate metabolism, namely sucrose phosphate synthase 1F (*SPS1F*), two of trehalase1 (*TRE1*), pectin methylesterase 3 (*PME3*), pectinesterase inhibitor 39 (*PEI39*), non-specific phospholipase C3 (*NPC3*), and chitinase-like protein (*CTL*). Some of these genes are involved in SRs development. *SPS1* is positively correlated with starch content in developing storage roots of cassava, and it likely plays a role in accelerating starch accumulation^66^. Trehalase enzyme (*TRE1*) hydrolyzes trehalose into glucose molecules. High trehalose content in *Attre1* knockout plant reduced both the vegetative and reproductive growth of Arabidopsis^67^. Perturbation of trehalose metabolism in potato hindered tuber formation and production^36^. *NPC3* encodes the phosphatidylcholine-hydrolyzing phospholipase C enzyme. *NPC3* knockout in Arabidopsis resulted in low lateral root density^68^. In addition, seven TFs were identified as DNB genes, namely telomere repeat-binding factor 2 (*TBP2*), MYB-like transcription factor (*ETC1*), OBF binding protein 4 (*OBP4*), indeterminate (ID)-domain 5 (*IDD5*), DNAJ homolog superfamily C member 17 (*DNAJC17*), WRKY DNA-binding protein 32 (*WRKY32*) and *ERF*. *OBP4* shows negatively regulated lateral root development by inhibiting the nitrate-responsive *XTH9* gene^69^. MYBs and WRKYs families are involved in regulating vascular cambium activity required for secondary cell growth of SR^10^. The ethylene signaling *ERF* gene regulates cassava SR initiation^14^.

**Figure 5:**
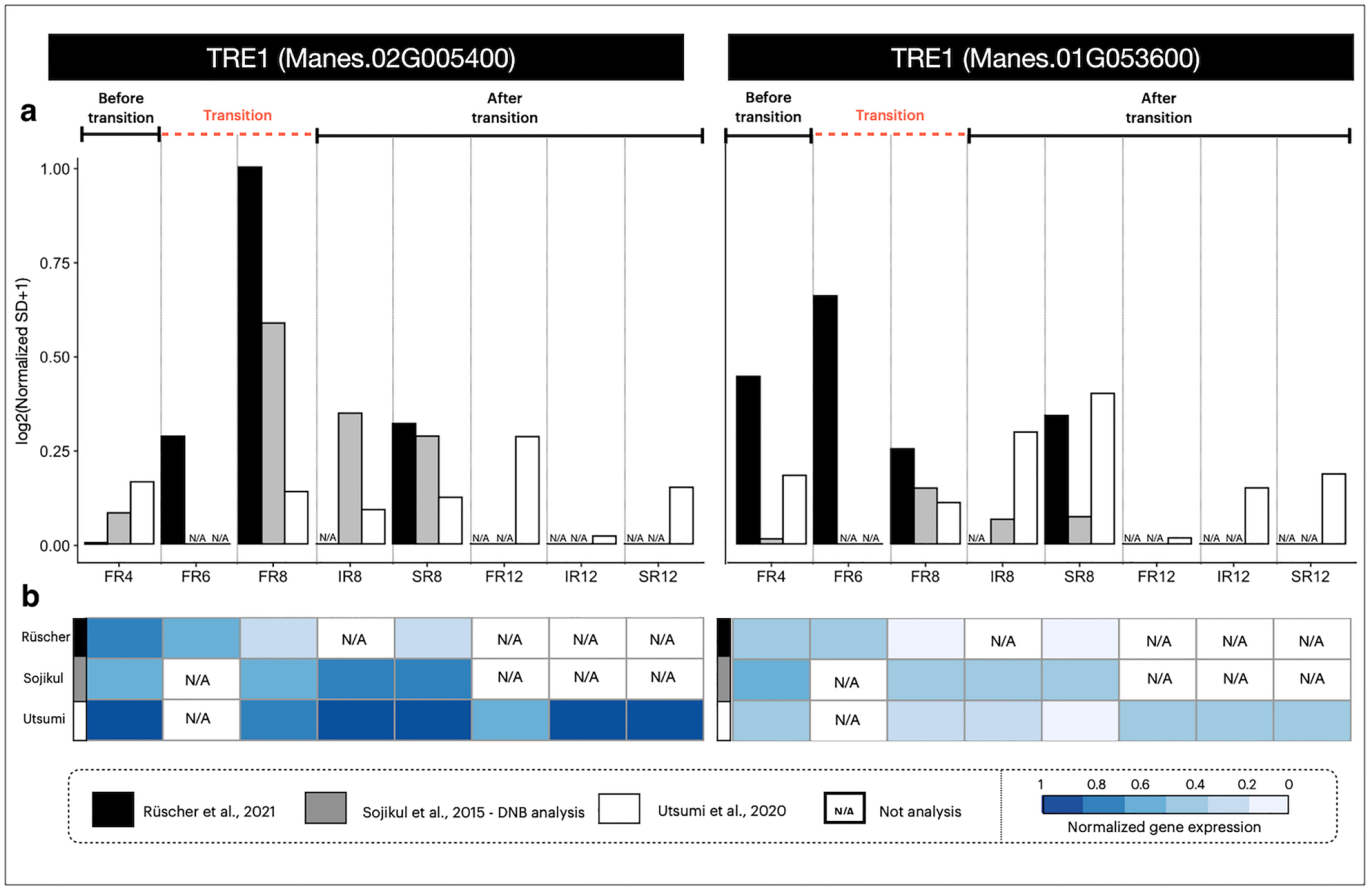
DNB characteristics of trehalase gene (*TRE1*) expression during cassava storage root development in two independent transcriptome datasets, GSE143278^15^ and PRJEB41121^13^. (**a**) Gene expression variance of *TRE1* in replicated samples. (**b**) Gene expression pattern of *TRE1* during SR development. Colors represent levels of gene expression from low (white) to high (blue). N/A denotes “data not analyzed”. FR, IR, and SR denote fibrous, intermediate and storage roots, respectively, at 4, 8 and 12 weeks after planting, as indicated.

Functional analysis by gene ontology (GO) term enrichment suggests the DNB genes’ involvement in regulating SR initiation using different regulatory mechanisms. Two enriched GO terms were linked to the trehalose catabolic process (GO:0005993) and alpha, alpha-trehalase activity (GO:0004555) (Fig. 4b). Interestingly, both enriched GO terms pointed to *TRE1* genes (Manes.01G053600 and Manes.02G005400), demonstrating the importance of trehalose metabolism for cassava SR initiation. *TRE1* and genes in trehalose metabolism were further investigated with two independent transcriptome data on early developing SR, GSE143278^15^ and PRJEB41121^13^, in order to study their roles in modulating transition of SR. Figure 5 showed that the *TRE1* gene consistently expressed DNB character in all transcriptome data. *TRE1* exhibited a similar expression pattern across all root development stages, with a high replicate variability of fibrous roots at approximately 6–8 WAP. By contrast to *TPS* and *TPP* genes, *TRE1* showed DNB character across the three independent datasets^13–15^ (Supplementary Fig. S2). The expression of trehalose metabolic genes (*TPS*, *TPP*, and *TRE1*) typically exhibits greater variability across biological replicates (Supplementary Fig. S3), reflecting the truly dynamics in their gene expression. Additionally, our analysis revealed that the variation in these metabolic genes among replicates is usually greater than that observed across developmental stages (Supplementary Fig. S4), suggesting the nature of the trehalose metabolism gene expression. However, the trehalose genes performed similarly in these datasets, but more especially in the study of Rüscher et al.^13^, suggesting their involvement at the early stage of SR development. Our findings suggest that trehalose genes, especially *TRE1*, play a key role in regulating the transition from FR to SR, probably by interconnecting the central growth regulation and carbon metabolism through patterning the T6P cumulative levels in similar manner to *TPS* and *TPP* genes as observed in potato^36^. The cooperative regulatory manner of *TRE1* was also observed in budding yeast where its action links carbon metabolism to cell division^70^.

### Trehalose metabolism possibly coordinates transcriptional regulation and carbon metabolism to determine fibrous-storage root transition in cassava

**Figure 6:**
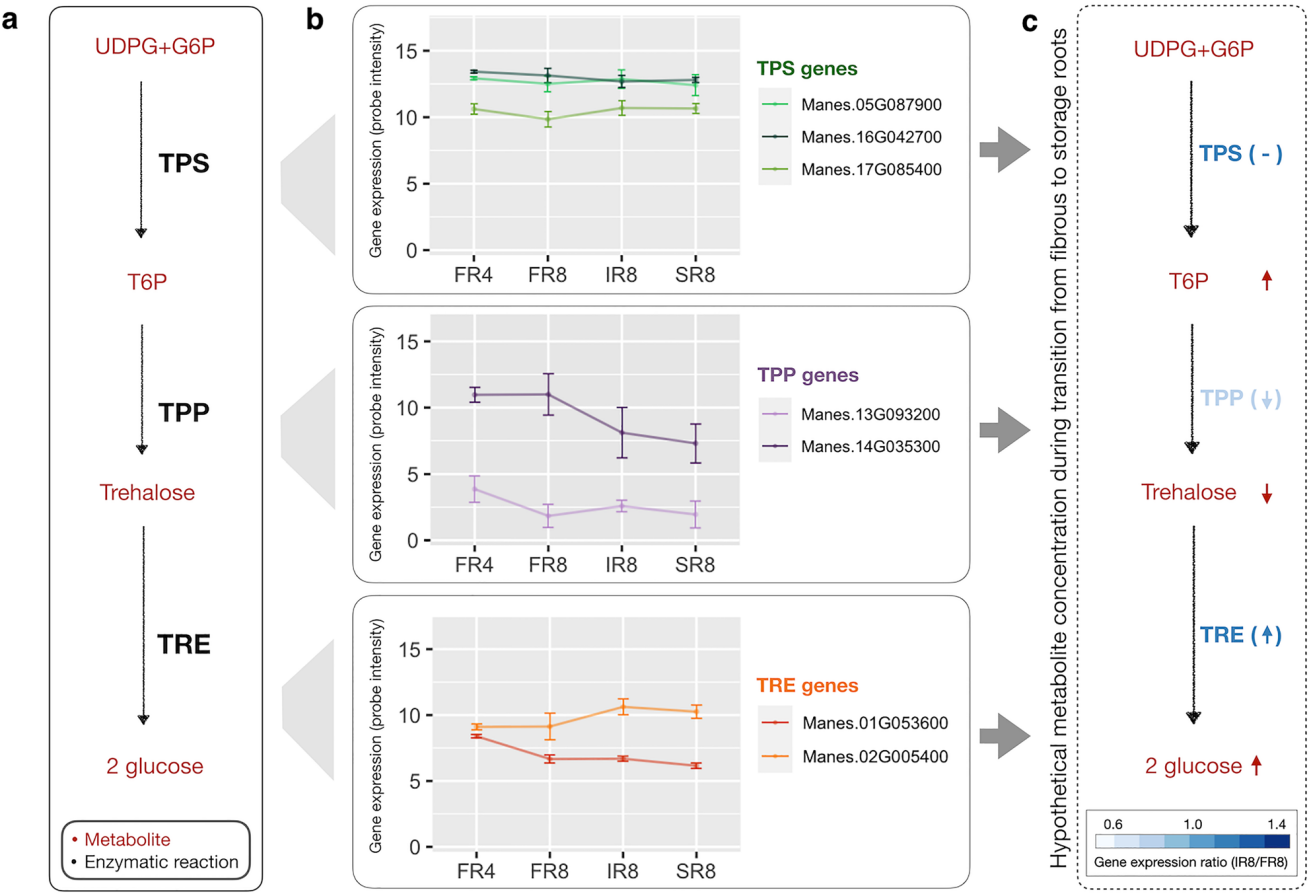
The expression profile of genes encoding enzymes involved in trehalose metabolism associated with SR initiation. (**a**) Trehalose metabolism covers the conversion of uridine diphosphate-glucose (UDPG) and glucose-6-phosphate (G6P) to glucose molecules, catalyzed by trehalose-6-phosphate synthase (TPS), trehalose-6-phosphatase (TPP) and trehalase (TRE). (**b**) The expression profile of trehalose related genes. X-axis denotes the stage of SR initiation. FR4 denotes fibrous roots of four-weeks-old plants. FR8, IR8 and SR8 denote fibrous, intermediate, and early storage roots of 8-weeks-old plants. Y-axis denotes gene expression by microarray probe intensity. (**c**) The hypothetical metabolite concentration based on gene expression profiles during the transition from FR8 to IR8. The color for each enzymatic gene represents the fold change in the expression of IR8 relative to FR8. Blue arrows denote a hypothetical tendency of enzymatic activity in the pathway. Red arrows represent a hypothetical tendency of metabolites level in the pathway.

To further understand how trehalose metabolism links cellular growth with carbon metabolism to regulate the transition from fibrous to storage roots, we studied the expression profiles of seven genes encoding key enzymes in trehalose metabolism during cassava SR initiation. Trehalose metabolism is involved in the conversion of uridine diphosphate-glucose (UDPG) and glucose-6-phosphate (G6P) into glucose molecules, catalyzed by trehalose-6-phosphate synthase (TPS), trehalose-6-phosphatase (TPP), and trehalase (TRE) (Fig. 6a). The expression of genes in trehalose metabolism changed in association with SR formation. Three genes encoding TPS were highly expressed across all stages of SR development (Fig. 6b), while two *TPP* genes declined with increased *TRE1* expression in bulking SR (Fig. 6b). According to the enzymatic gene activity, the conversion of T6P to trehalose tended to decrease during the transition to storage roots. Maintaining a high T6P concentration and a low trehalose concentration help prevent trehalose toxicity to growth (Fig. 6c).

**Figure 7:**
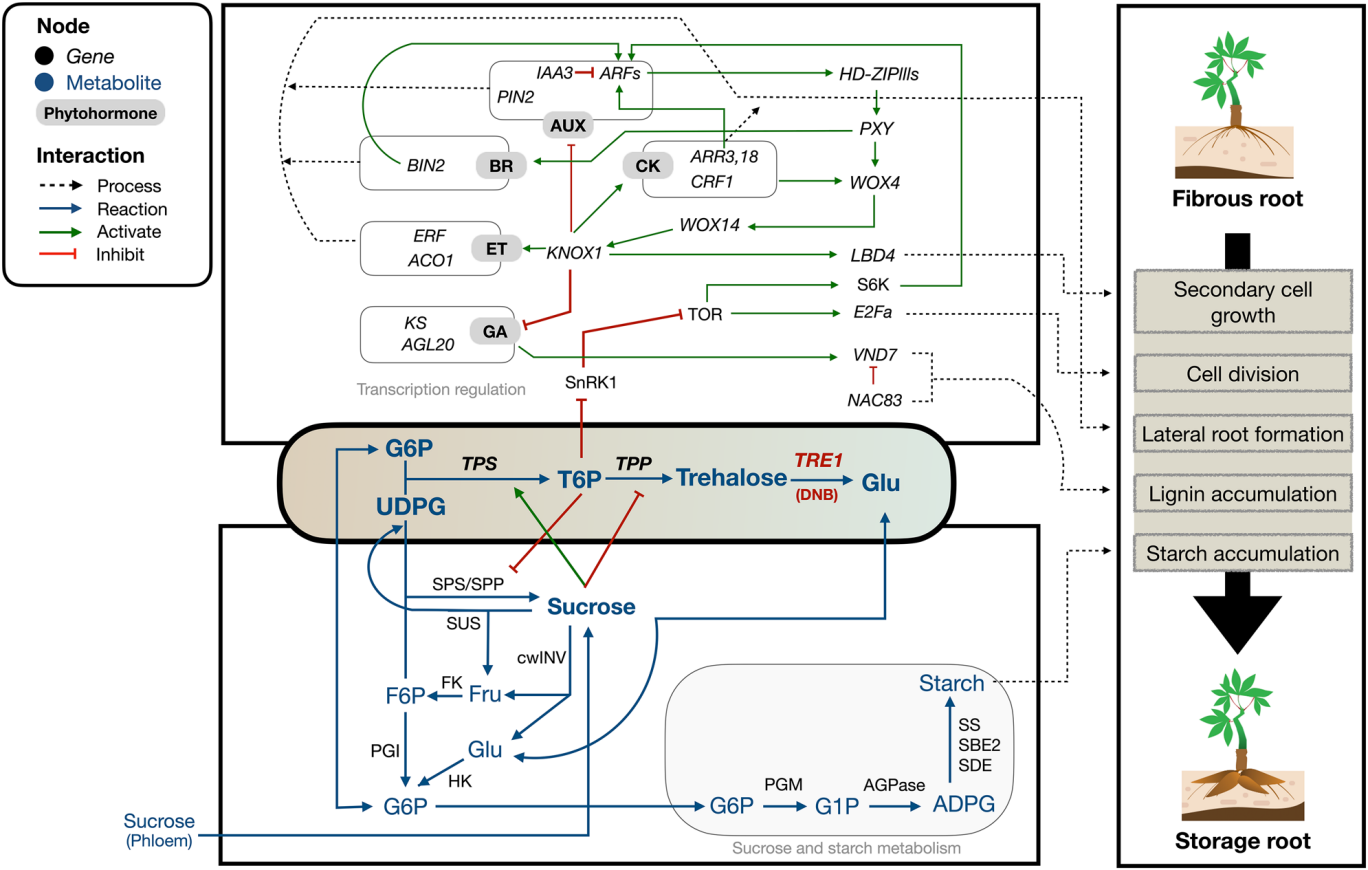
Schematics of the gene regulatory model indicating potential roles of trehalose metabolism in modulating SR initiation.

Trehalose metabolism helps regulate sucrose levels, plant growth and carbon status, crucially regulating bulking, the transition from fibrous to storage roots. Inclusive integration of the analyzed results and evidence existing in the literature (Supplementary Data S3) allowed the proposition of trehalose metabolism in action by linking regulatory control and carbon metabolism for root development (Fig. 7). The abundance of sugar metabolites in trehalose metabolism likely acts as a signal to synchronize transcriptional regulatory control and carbon metabolism. During the transition (FR8–IR8) period at the eighth week, *TPP* expression in FR8 declined (Fig. 6b) in response to root cell sucrose unloading, leading to the rising level of T6P, which coincided with the T6P-sucrose nexus model that links sucrose to increased TPS and reduced TPP activity^32^. The high T6P level then suppresses SnRK1 and activates TOR, a central growth regulator involved in plant growth and linked to phytohormones^33,35,71^. TOR activity induces E2Fa, a key player in the cell cycle and cell division, and also activates S6K, which subsequently promotes auxin-related gene activation (*ARFs*)^33,72^, leading to *KNOX1* activation. *KNOX1* promotes various downstream regulatory pathways such as phytohormone-related regulation (*ARR3*, *ARR18*, *CRF1*, *BIN2*, *ERF* and *ACO1*) for lateral root formation^14,15^ and secondary cell growth pathways by activating vascular cambium-related genes (*HD-ZIPlll*, *PXY*, *WOX4*, *LBD4*)^10,13^. Additionally, *KNOX1* inhibits gibberellic acid (GA) related genes (*KS* and *AGL20*), which activate *VND7* involved in the lignification process^21,73,74^. Overall, it suggested *TPS* and *TPP* actions are likely more relevant to T6P patterning for storage root growth (Fig. 7) as also corresponding to those summarized by Fichtner et al.^34^ and Schluepmann et al.^75^.

To achieve SR bulking, the concentration of sugars in trehalose metabolism may change to support starch biosynthesis and filling. The negative feedback regulation of T6P-sucrose nexus, may operate as a driving force in the starch-induction process. High T6P levels in turn decreased the SPS and SPP activity which may exert pressure on the cell to enhance the conversion of sucrose to starch via activating another starch synthesis process. *TRE1* was highly expressed at transition, and its expression continuously increased after the transition (Fig. 6b). *TRE1* is thus presumed to regulate SR transition by adjusting the abundance of metabolites (i.e., T6P, trehalose, sucrose and glucose) and balancing sugars to induce starch synthesis, as also suggested in *Attre1* loss of function mutant in Arabidopsis plant^67^. TRE1 hydrolyzes trehalose into glucose, which is then converted into G6P by HK. This process promotes starch production, activates starch and sucrose metabolism, and optimizes SR growth by preventing trehalose toxicity^76–78^. The mechanistic process is well-studied in Arabidopsis model plant, and is believed to be involved in governing growth and development in various plant species. There was high expression of genes encoding key enzymes involved in sucrose and starch syntheses such as *SuSy*, *FK*, AGPase of the small (*APS*) and large (*APL*) subunits, and *SBE*, after the transition, corresponding to *TRE1* (Supplementary Fig. S5). Combining the current knowledge with our analysis, the results suggested that *TRE1* may modulate T6P patterns to signal growth stage transitions, in contrast to *TPS* and *TPP*, which are thought to primarily influence T6P patterns for developmental growth. Taken together, the model proposed herein demonstrates a potential link between trehalose metabolism and the regulation of SR initiation.

## Discussion

Transitioning from fibrous to storage roots is crucial for cassava root yield enhancement. Identification of key regulators of storage root initiation is challenging for several reasons. First, the initial stage of storage root bulking is hard to capture. Anatomical and physiological studies have mainly focused on secondary cell growth of phloem, xylem via the activity of vascular cambium^6,14^. The onset of SR initiation could vary during the first 3 MAP depending on the cultivar and environment^5^. Second, timely regulatory signals and sufficient carbon substrates are supposed to be well coordinated to begin the transition to SR. However, research has mainly focused on gene expression analysis during SR development, largely neglecting the association with both processes. Decades of studies based on genome-wide gene expression analysis have identified genes involved in SR development^10,13–15,27,79–81^, yet the actual regulators of transition remain investigated. Recent studies have offered some insights into the importance of timely gene regulation at the early stage of SR development^13–15^. Candidate genes such as *Mec1*, *RING Zinc Finger* and *TCTP*, highly expressed in SR than FR^79–81^, *KNOX1*, *ERF*, *ARR3*, *BIN2*, *AGL20* and *ARFs*, associated with phytohormones^14,15^, and *PNY*, *PXY*, *WOX4*, and *LBD4*, transcriptional activators of secondary growth factors^13^. One limitation of the DEG analysis is that it ignores genes showing highly variable expression among individual samples, a characteristic behavior of regulators at the early stage of transition^43,44^. Through DNB analysis, we show that trehalose metabolism, especially *TRE1*, is potentially a key regulator of FR-to-SR transition. Trehalose metabolism helps coordinate endogenous regulatory signaling and carbon substrate availability through the connection of transcriptional regulation and sugar metabolism.

Gene expression changed globally and dynamically during the development of SR, from FR4 to SR8. Analysis of overall gene expression suggested that the transition period to SR happened around 6–8 WAP (FR8) (Figs. 1 and 2). Gene expression at FR8 was highly variable between samples, typical for systems under transition, according to the bifurcation theory^43,44^. The gene expression profile of IR was similar to SR, which reflected their morphological and anatomical similarities in terms of high cell expansion and starch accumulation^5,6^. FR initiates the differentiation of primary into secondary tissues and develops starch-specific organelles (starch granules), visible in both IR and SR^6^. There were 99 key regulators proposed to modulate the transition from fibrous to storage roots. These genes were highly regulated and showed expression characters distinct from the SR-related genes identified by DEG (Fig. 3). Among the 99 DNB genes, we identified those related to TFs, phytohormones, secondary cell growth, and carbohydrate metabolism (Supplementary Data S1). These processes are well-documented as playing a vital role in the formation of storage roots, where the coordination of TFs and phytohormones drives the active development of secondary cell growth and carbon metabolism for starch biosynthesis^10,13–15^. Interestingly, it was observed that DNB genes were mostly acted upstream of the SR-related genes proposed by DEG analysis. This was observation supported the interaction of the two regulatory gene sets, with DNB upstream of DEG genes^52^. In sweet potato, the upstream DNB gene *IbNAC083* helps initiate root swelling by regulating the expression of several DEG genes (DEG) linked to lignin and flavonoid biosynthesis^20^. The functional analysis of DNB genes also reinforced their role in SR initiation and showed connections to phytohormones and carbon metabolism (Fig. 4a), which stimulate cell proliferation and starch accumulation during cassava SR formation^10,12–15,18–20,27^. Moreover, the importance of trehalose metabolism in regulating FR transition to SR by coordinating the endogenous regulatory control and carbon metabolism, as reported in potato^36^ was highlighted (Fig. 4b). Dabest et al. showed that altering *TPS* and *TPP* expression, thereby changing the level of metabolites in trehalose metabolism, especially T6P, affected both tuber growth and carbohydrate content, supporting its role in linking plant growth regulatory pathway and sugar metabolism^36^.

Trehalose metabolism has been investigated in a wide range of plants, including Arabidopsis^75^, rice, wheat, maize, sorghum^82^ and potato^36^, but never in cassava nor about its coordination of developmental stage transition. Trehalose metabolism is regulated by three enzymes corresponding to 7 genes, *TPS* (Manes.05G087900, Manes.17G085400 and Manes.16G042700), *TPP* (Manes.13G093200 and Manes.14G035300) and *TRE1* (Manes.02G005400 and Manes.01G053600 that are responsible for controlling the levels of intermediate sugars, such as T6P and trehalose, in this pathway. The expression of trehalose metabolic genes was shown highly correlated with their encoded enzymatic protein activity^77,83^. Abundance of T6P is controlled by *TPS* and *TPP*^33,34^. For instance, loss of *AtTPS1* function can change the T6P level, affecting flowering and embryonic morphogenesis transition in Arabidopsis^84,85^. Loss of the *ra3* gene encoding TPP enzyme affects axillary meristem development in maize^86^. Alteration of *TPS* and *TPP* expression changes the level of T6P, affecting the growth and carbohydrate content in tuberous roots of potato^36^. High T6P and sucrose accumulation was found at the onset of seed filling in pea^87^, similar to the pattern of metabolites in this study (Fig. 6). Loss of *AtTRE1* function can disrupt vegetative growth, reproductive organ development and carbon metabolism by affecting the expression of sucrose and starch metabolic genes in Arabidopsis^67^. Our analysis showed the role of DNB gene *TRE1* in regulating SR initiation, as indicated by its high expression profile during the SR initiation and high trehalose conversion to glucose for starch accumulation. Other studies similarly reported high expression of sucrose and starch-related genes at the early stage of SR development^88^, and glucose conversion to G6P for starch biosynthesis^89^. Low levels of trehalose were detected in many plants, and it was suggested the high activity of the trehalase enzyme would prevent trehalose toxicity and allow for plant development^76,90,91^. Similar observations have supported that *TRE1* plays a crucial role in reducing trehalose toxicity and maintaining the balance of soluble sugar and starch in both the aboveground and belowground of plants via the overexpression of *SnRK1*^78^. Increasing trehalose accumulation by inhibiting *TRE1* expression alters sucrose and starch contents in Arabidopsis and suppresses reproductive growth compared to wild-type^67^. *TRE1* regulates metabolites for optimized growth and sucrose supply together. Inhibition of TRE1 activity by validamycin A reduced sucrose and starch in Arabidopsis tissues, indicating TRE1’s role in plant carbohydrate allocation^77^. In budding yeast, *TRE1* helps coordinate proteins linked to cell division and metabolism by converting trehalose into sugars for serving central carbon metabolism during G1/S transition^70^. Our findings proposed the role of trehalose metabolism, particularly *TRE1*, in regulating the transition of SR through interconnecting central growth regulation and carbon metabolism via modulating the cumulative levels of T6P. The regulation is supposed to be in a similar manner to those observed in potato^36^.

Overall, this work highlighted the coordinating regulatory processes associated with SR initiation. Metabolic genes often show low expression levels relative to other genes in the genome, makes then hard to detect by the typical DEG analysis. Analysis of fluctuations in gene expression of highly associated groups of genes, using the DNB approach, identified novel regulators that govern the transition stage of cassava SR initiation. Our analysis suggested the linkage between transcriptional regulation and sugar metabolism in initiating the transition from FR to SR. The proposed hypothetical model was rigorously analyzed with extensive transcriptome data and the current ground evidence in cassava and other plant species (Fig. 7). While the results are certainly promising, it is crucial to conduct experimental validation when practical measurements become feasible. Finally, further research into the post-transcriptional and post-translational mechanisms is recommended.

## Conclusions

This study sheds new light on the regulation that underlies the transition from FR to SR during cassava SR initiation. Using DNB analysis, the transition stage and key regulators governing the phase transition were successfully identified, results that had proven elusive with the typical differential gene expression analysis. The association of the identified DNB genes with SR initiation was demonstrated. We showed the relevance of trehalose metabolism and, in particular, the role of *TRE1* as a key regulator and a timely regulatory signal that coordinates transcriptional regulation and sugar metabolism to reach the onset of SR initiation.

## Methods

### Gene expression data and analysis

Microarray gene expression data of cassava roots retrieved from Sojikul et al.^14^, were used here to identify the key regulator modulating the transition stage of cassava storage root initiation. The study was performed in KU50 high-yielding cassava cultivar. The genome-wide gene expression data contained triplicates of four data-points from four stages of root development: fibrous roots at four WAP (FR4), fibrous, intermediate and storage roots at eight WAP (FR8, IR8 and SR8, respectively). For data pre-processing, homology search with Bi-directional BLAST nucleotides (BLASTn) was used to re-annotate the 60-mer-oligo microarray probes on the cassava reference genome AM560 version 6.1 provided by the Phytozome database^92^. The probes were assigned to cassava genes at E-value ≤ 1e−10 and identity percentage ≥ 95% in both BLAST directions. The expression value of multiple probe sets corresponding to the same gene was also included in this study by selecting a representative probe with the following criteria: the best score of E-value, identity percentage, and minimum average standard deviation (*SD*) across all samples. Principal component analysis (PCA) was performed on the gene expression data to investigate the expression profile during the developmental stages of cassava roots, using the built-in prcomp function in R software (version 4.2.1). Unsupervised hierarchical clustering was applied to cluster the microarray samples by using Euclidian distance as default in pheatmap R package (version 1.0.12). The coefficient of variance (*CV*) was calculated for three biological replications at each root stage to study the gene expression variation during SR initiation. All visualization was achieved by using ggplot2 (version 3.3.6) and ggthemr (version 1.1.0) packages in R software.

Two independent transcriptome datasets: GSE143278^15^ and PRJEB41121^13^ were retrieved from the National Center for Biotechnology Information (NCBI) database for verification of the expression of trehalose metabolic genes obtained from DNB analysis. Each individual datasets were normalized and analyzed with respect to the overall gene expression in their own conditions. The microarray gene expression data of cassava KU50 at FR4, FR8, FR12, IR8, IR12, SR8 and SR12 stages (GSE143278) were pre-processed as aforementioned. PRJEB41121, the RNA-Seq data of cultivar TME419, was first assessed for the quality of raw reads with FastQC version 0.11.9. The adaptor sequences and low-quality bases were trimmed using Trimmomatic version 0.39. The cleaned reads were aligned to the cassava reference genome AM560 version 6.1 using STAR version 2.7.8a. The count reads were normalized by using Gene length corrected trimmed mean of M-values (Ge-TMM)^93^ before further analysis. Genes with expression values of log2(GeTMM) + 1 ≤ 0 in at least one sample were filtered out. The resulting relative expression patterns and variances at FR4, FR6, FR8, FR12, IR8, IR12, SR8 and SR12 were compared to examine the consistency among all studies.

### Dynamic network biomarker (DNB) analysis

DNB genes for cassava root developmental stages during SR initiation were identified following Chen et al.^43^. For each stage of cassava root development, the candidate genes with significant gene expression variation among biological replicates, based on a student t-test with Benjamini and Hochberg correction (FDR) and fold-change of standard deviation (FC_SD_) relative to control (FR4), were firstly selected. The criteria of FDR and FC_SD_ (FDR_FR8_ ≤ 0.72, FDR_IR8_ ≤ 0.20, FDR_SR8_ ≤ 0.25 with FC_SD_ ≥ 2) were based on Sojikul et al.^14^ and Chen et al.^43^, respectively. Secondly, the candidate genes in each stage were clustered using hierarchical clustering with optimized K = 80 as a cutoff (Supplementary Fig. S6), resulting in 80 clusters at each stage, namely FR8, IR8 and SR8. Thirdly, the three properties of DNB, namely $\it \it {\text{SD}}_{\text{in}}$, $\it \it \left|{\text{PCC}}_{\text{in}}\right|$, and $|{PCC}_{out}|$, were calculated for each cluster for FR8, IR8 and SR8. Clusters with the highest scores of $\it \it {\text{SD}}_{\text{in}}$, and $\it \it \left|{\text{PCC}}_{\text{in}}\right|$ and the lowest score of $|{PCC}_{out}|$ were considered representative of the complete DNB properties. Composite index ${(CI)}$ scores were calculated for the representative clusters as shown in Eq. (1):

1 $$\text{Composite index (CI)}= \frac{{\text{SD}}_{\text{in}} \times |{\text{PCC}}_{\text{in}}|}{|{\text{PCC}}_{\text{out}}|}$$

where, $\it \it {\text{SD}}_{\text{in}}$ denotes the average standard deviation of gene expression within a cluster, $\it \it \left|{\text{PCC}}_{\text{in}}\right|$ denotes the average Pearson correlation coefficient (*PCC*), in absolute value, within a cluster and $|{PCC}_{out}|$ denotes the average Pearson correlation coefficient (*PCC*) between clusters.

Lastly, *CI* scores of all representative clusters were compared, and the cluster with the highest $\it {{CI}}$ score was proposed as a DNB cluster. The gene members of a DNB cluster were identified as key regulators modulating the phase transition, SR initiation. The stage corresponding to the DNB cluster was also identified as the transition stage, the onset of SR initiation.

### Construction and visualization of DNB and DEG-derived SR-related genes association network

To investigate the relationships between the DNB and DEG genes^10,13–15^ using the cassava transcriptional regulatory network (TRN) in the PlantRegMap database as a reference network^58^. Nodes in the constructed network indicate DNB and DEG-derived SR-related genes whose linkages are found in PlantRegMap, and edges indicate interactions between DNBs and first-order neighboring DEG genes based on the cis-regulatory element. To further characterize the gene association network, the gene expression ratio and standard deviation (*SD*) ratio for FR8 and FR4 were calculated and visualized on a percentile rank scale. Network visualization was done by using Cytoscape version 3.9.1^94^.

### Functional analysis

Odds ratio estimation was done using the GeneOverlap package (version 1.26.0) in R software to study the association between the identified DNB and functional genes related to SR initiation and development collected from literature and databases. The genes linked to SR initiation and development comprised 1925 SR-related genes^10,13–15^, 445 phytohormone genes^13,15^, 929 carbohydrate metabolism genes^95^, 2267 TF genes^96–98^, 1392 secondary cell growth genes^10,13^ and 1498 kinase-related genes^98^. To further investigate the DNB genes enriched functions, gene ontology (GO) enrichment analysis was conducted via GOATOOLS. The Python library for GO analysis was based on a hypergeometric test^99^. The *p*-value adjustment by the Benjamini–Hochberg error correction method (FDR) ≤ 0.05 was applied to select significantly enriched GO terms. Visualization of the enriched GO terms was done using the ggplot2 package in R software.

### Supplementary Information

Dataset S1.

Dataset S2.

Dataset S3.

Supplementary Figures.

## Data Availability

The dataset information used for the analyses during the current study is available at the Gene Expression Omnibus (GEO) repository, accession number GPL14139. The associated expressed data is available from the corresponding author on request in the published article by Sojikul et al.^14^. All other data generated or analyzed during this study are included in the published articles of Utsumi et al.^15^ (accession number GSE143278) and Rüscher et al.^13^ (Accession Number PRJEB41121).
