# Supplementary material for: Trehalose metabolism coordinates transcriptional regulatory control and metabolic requirements to trigger the onset of cassava storage root initiation
Source: Sci Rep. 2023 Nov 15;13:19973. doi: 10.1038/s41598-023-47095-8 (PMC10651926; doi:10.1038/s41598-023-47095-8)
Supplement: Supplementary file 4 — Supplementary Figures. [file 41598_2023_47095_MOESM4_ESM.docx]

Trehalose metabolism coordinates transcriptional regulatory control and metabolic requirements to trigger the onset of cassava storage root initiation

Nattavat Sukko^1^, Saowalak Kalapanulak^1,2,3*^, and Treenut Saithong^1,2,3*^

^1^ Bioinformatics and Systems Biology Program, School of Bioresources and Technology and School of Information Technology, King Mongkut’s University of Technology Thonburi (Bang Khun Thian), Bangkok 10150, Thailand

^2^ School of Bioresources and Technology, King Mongkut’s University of Technology Thonburi (Bang Khun Thian), Bangkok, 10150, Thailand

^3^ Center for Agricultural Systems Biology, Systems Biology and Bioinformatics Research Group, Pilot Plant Development and Training Institute, King Mongkut’s University of Technology Thonburi (Bang Khun Thian), Bangkok 10150, Thailand.

^*^E-mail: saowalak.kal@kmutt.ac.th and [treenut.sai@kmutt.ac.th](mailto:treenut.sai@kmutt.ac.th)

**Supplementary Figures**

**
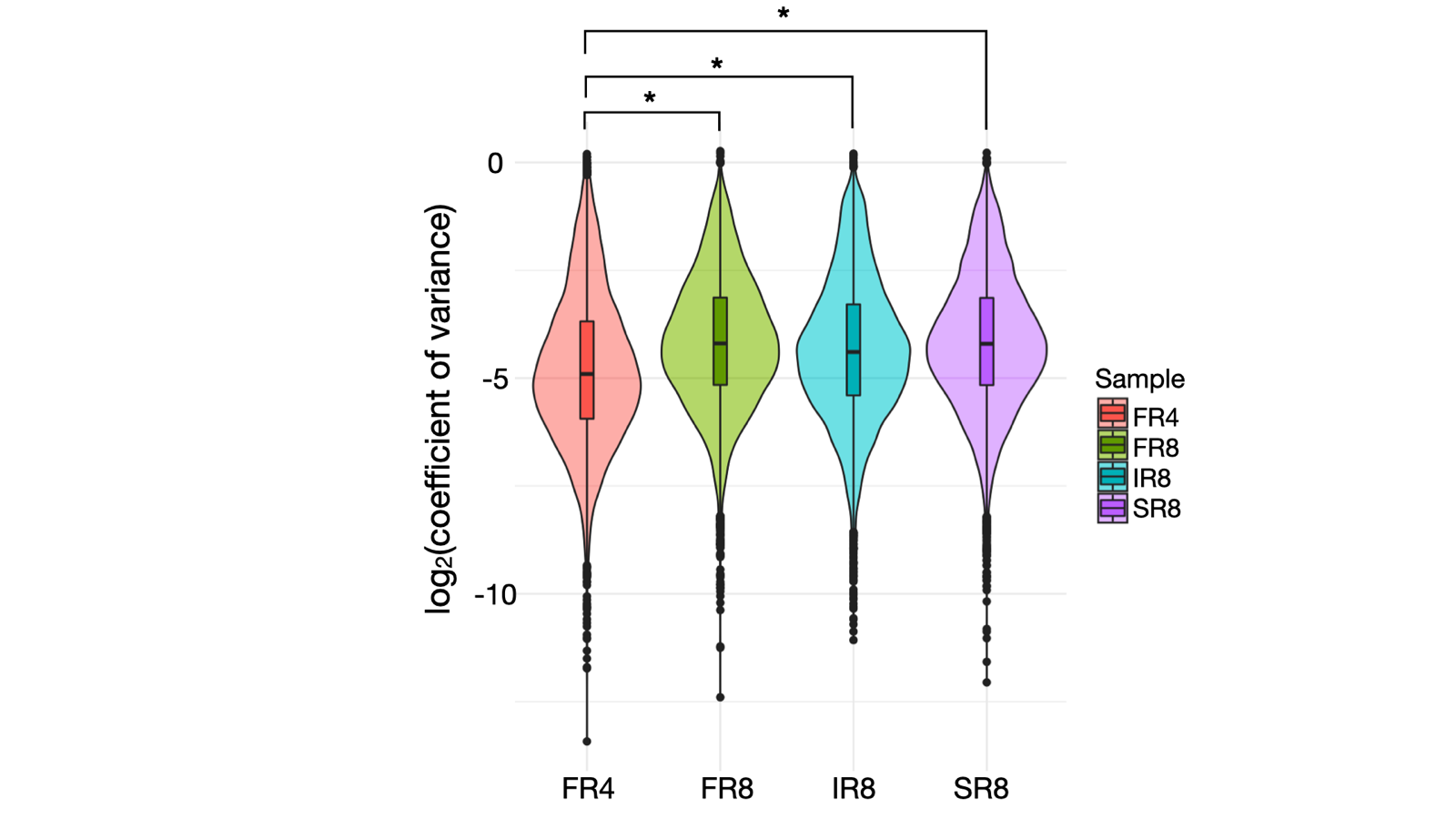
**

**Supplementary Figure S1.** Gene expression variance in the three biological replicates of root samples, FR4 - fibrous roots at four weeks after planting (WAP), FR8 – fibrous roots at eight WAP, IR8 – intermediate roots at eight WAP, and SR8 - storage roots at eight WAP. The asterisks denote the significantly higher variation than FR4 based on a one-tailed student t-test with *p*-value ≤ 0.05.


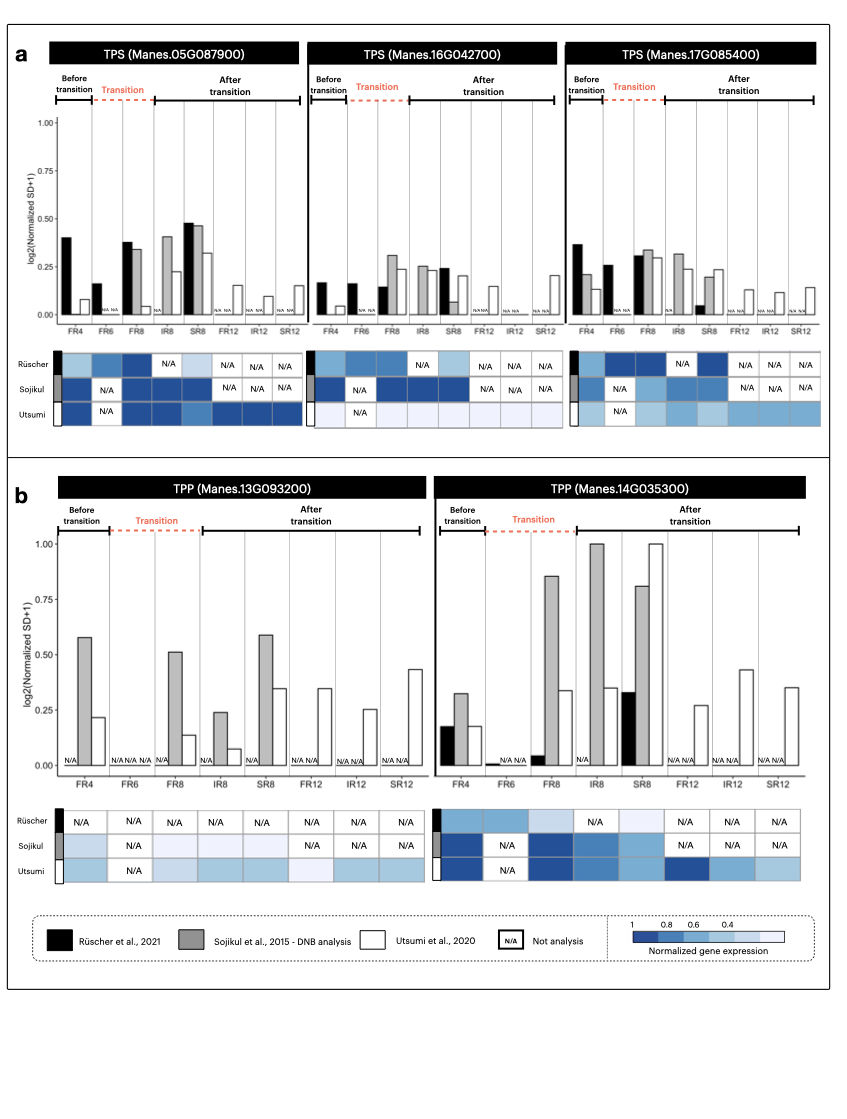


**Supplementary Figure S2.** The characteristics of **(a)** trehalose-6-phosphate synthase (*TPS*) and **(b)** trehalose-6-phosphatase (*TPP*) genes expression during cassava storage root development in two independent transcriptome datasets, GSE143278 ^[1]^ and PRJEB41121 ^[2]^. Top panel shows gene expression variance of **(a)** *TPS* and **(b)** *TPP* genes in replicated samples. Bottom panel shows gene expression pattern of **(a)** *TPS* and **(b)** *TPP* genes during SR development. Colors represent levels of gene expression from low (white) to high (blue). N/A denotes “data not analyzed”. FR, IR, and SR denote fibrous, intermediate and storage root, respectively, at four, eight and twelve weeks after planting, as indicated.


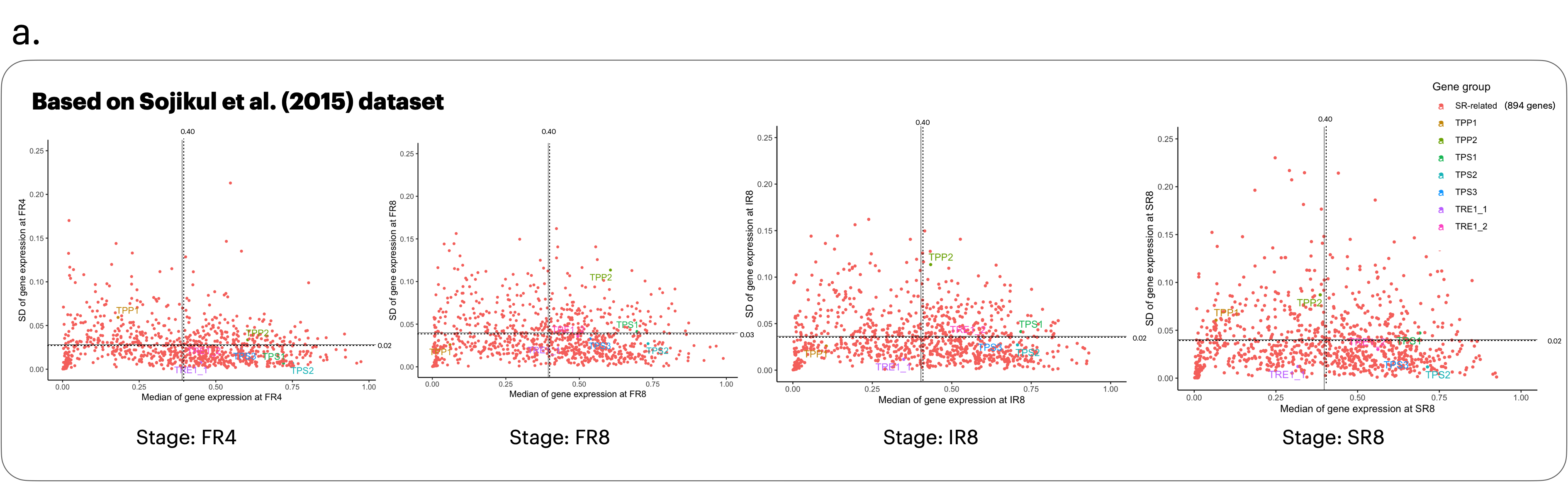


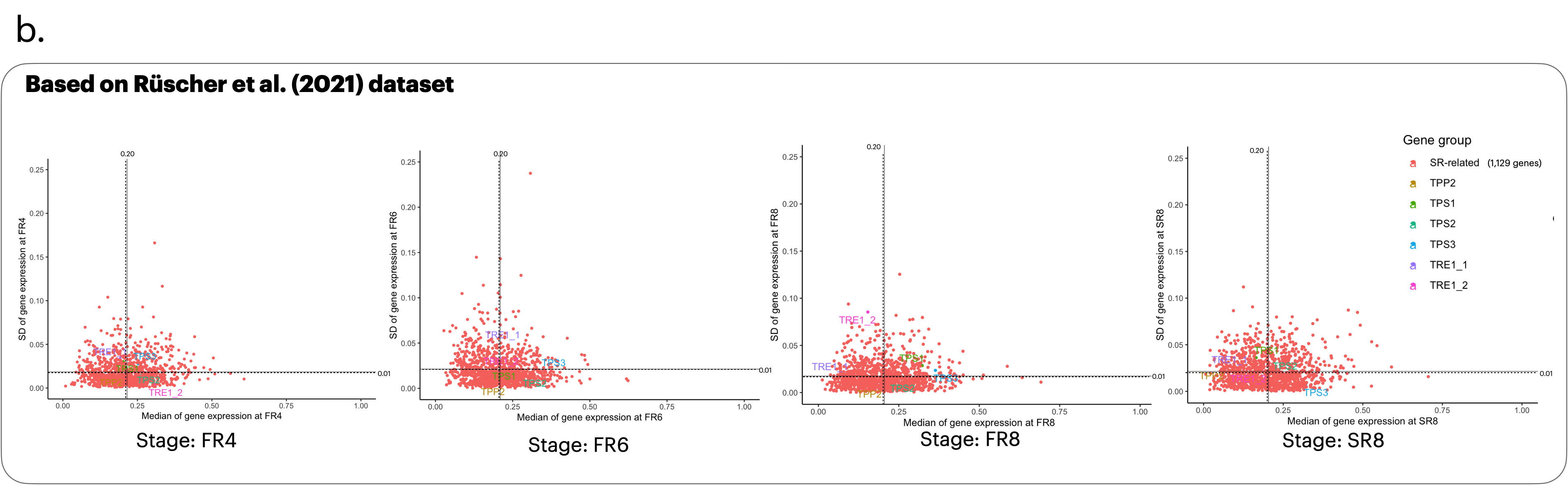


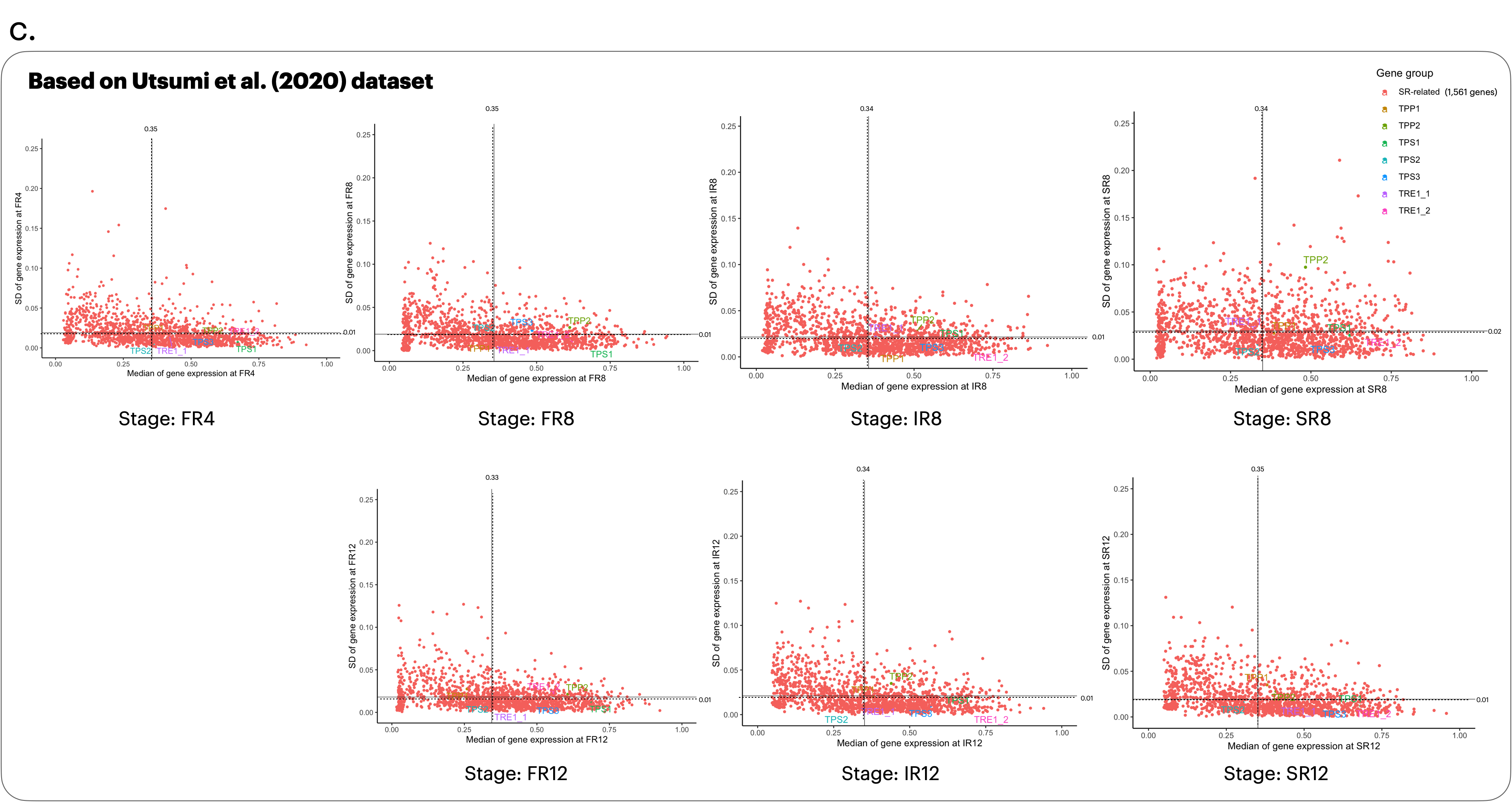


**Supplementary Figure S3.** Gene expression profiles of trehalose metabolic genes and SR-related genes from the three transcriptome datasets of cassava storage roots: (**a**) Sojikul et al. (2015), (**b**) Rüscher et al. (2021) ^[2]^, and (**c**) Utsumi et al. (2020) ^[1]^. The X-axis represents the median of gene expression, and the Y-axis represents the variation in gene expression among replicated samples (n=3). The solid and dash lines indicate the average expression level and variation based upon the SR-related gene expression and overall genes in the datasets, respectively. The colors represent gene groups, including trehalose metabolic genes (*TRE1_1* (Manes.01G053600), *TRE1_2* (Manes.02G005400), *TPS1* (Manes.05G087900), *TPS2* (Manes.16G042700), *TPS3* (Manes.17G085400), *TPP1* (Manes.13G093200) and *TPP2* (Manes.14G035300)) and SR-related genes. FR, IR, and SR denote fibrous, intermediate and storage roots, respectively, at four, eight and twelve weeks after planting, as indicated. All expression data was normalized by using min-max normalization in each dataset individually.


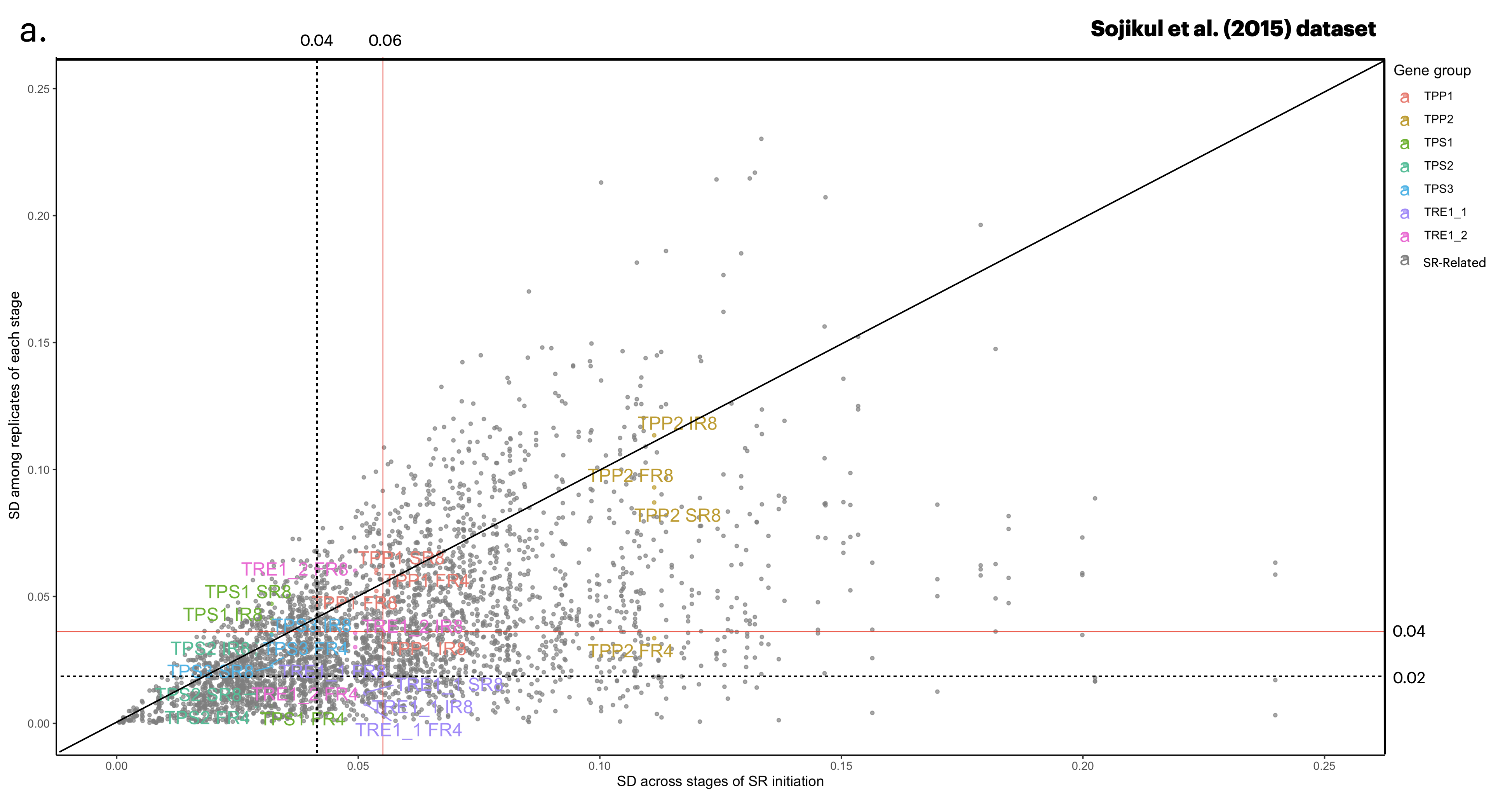


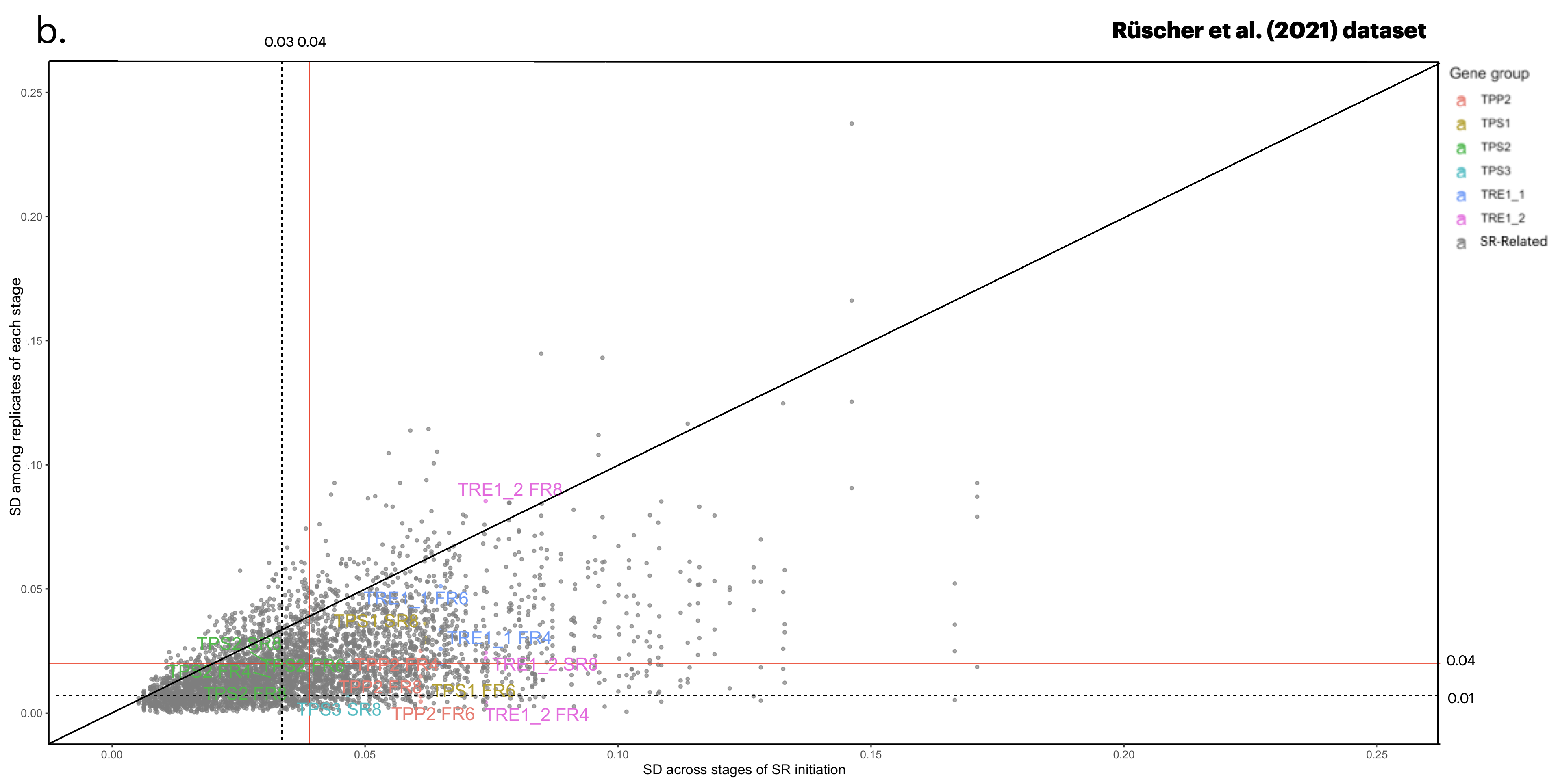


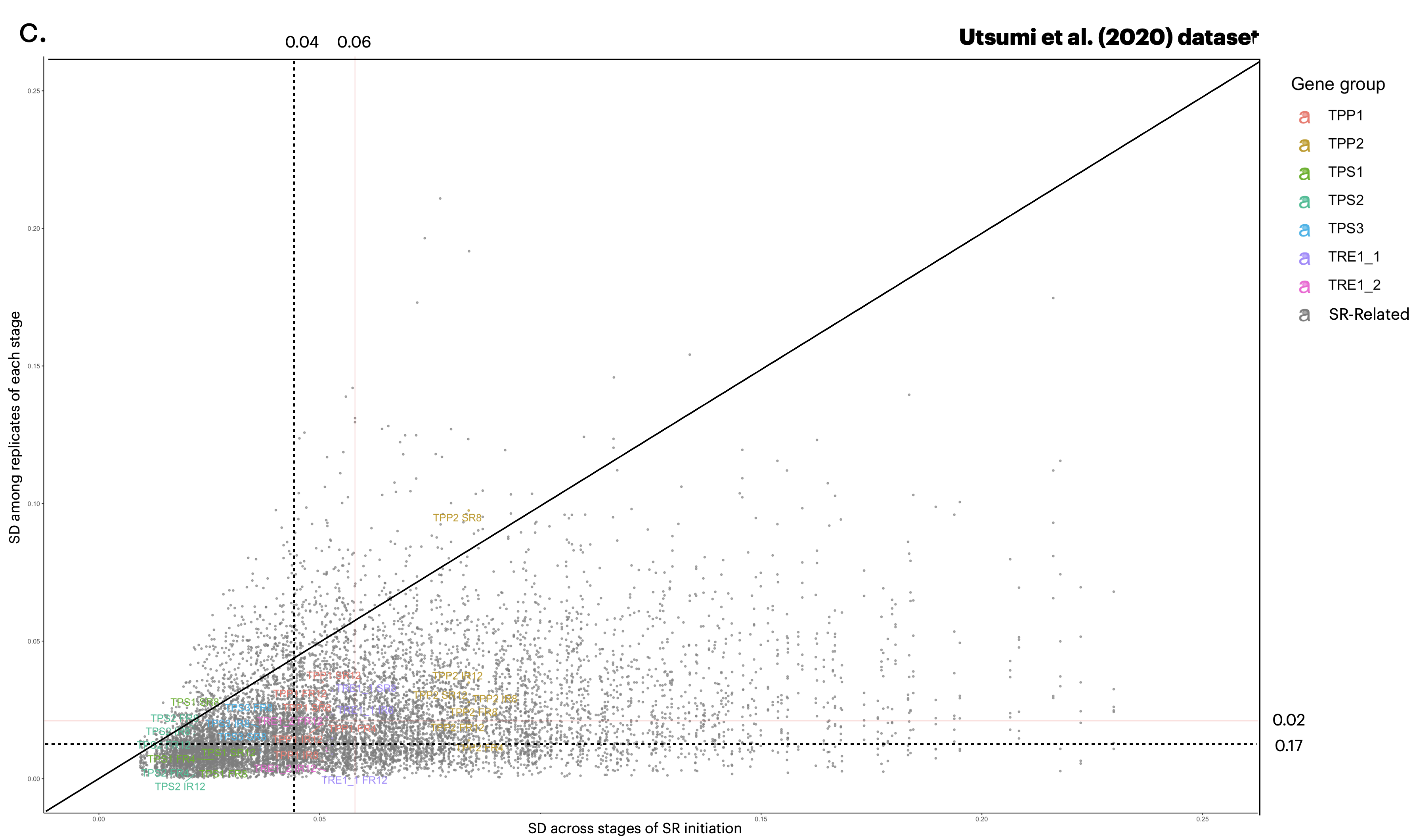


**Supplementary Figure S4.** The gene expression variation of T6P-related genes (*TPS*, *TPP* and *TRE1*) and SR-related genes from the three transcriptome datasets of cassava storage roots: (**a**) Sojikul et al. (2015), (**b**) Rüscher et al. (2021) ^[2]^, and (**c**) Utsumi et al. (2020) ^[1]^. The X-axis represents the variation in gene expression of all across in the developmental stages in each dataset, and the Y-axis represents the standard deviation (SD) among biological replicate of each stage of SR initiation. The solid and dash lines indicate standard deviation (SD) among biological replicate of each stage and all across in the developmental stages based upon the SR-related gene expression and overall genes in the datasets, respectively. The colors represent gene groups, including trehalose metabolic genes (*TRE1_1* (Manes.01G053600), *TRE1_2* (Manes.02G005400), *TPS1* (Manes.05G087900), *TPS2* (Manes.16G042700), *TPS3* (Manes.17G085400), *TPP1* (Manes.13G093200) and *TPP2* (Manes.14G035300)) and SR-related genes. All expression data was normalized by using min-max normalization in each dataset individually.

**
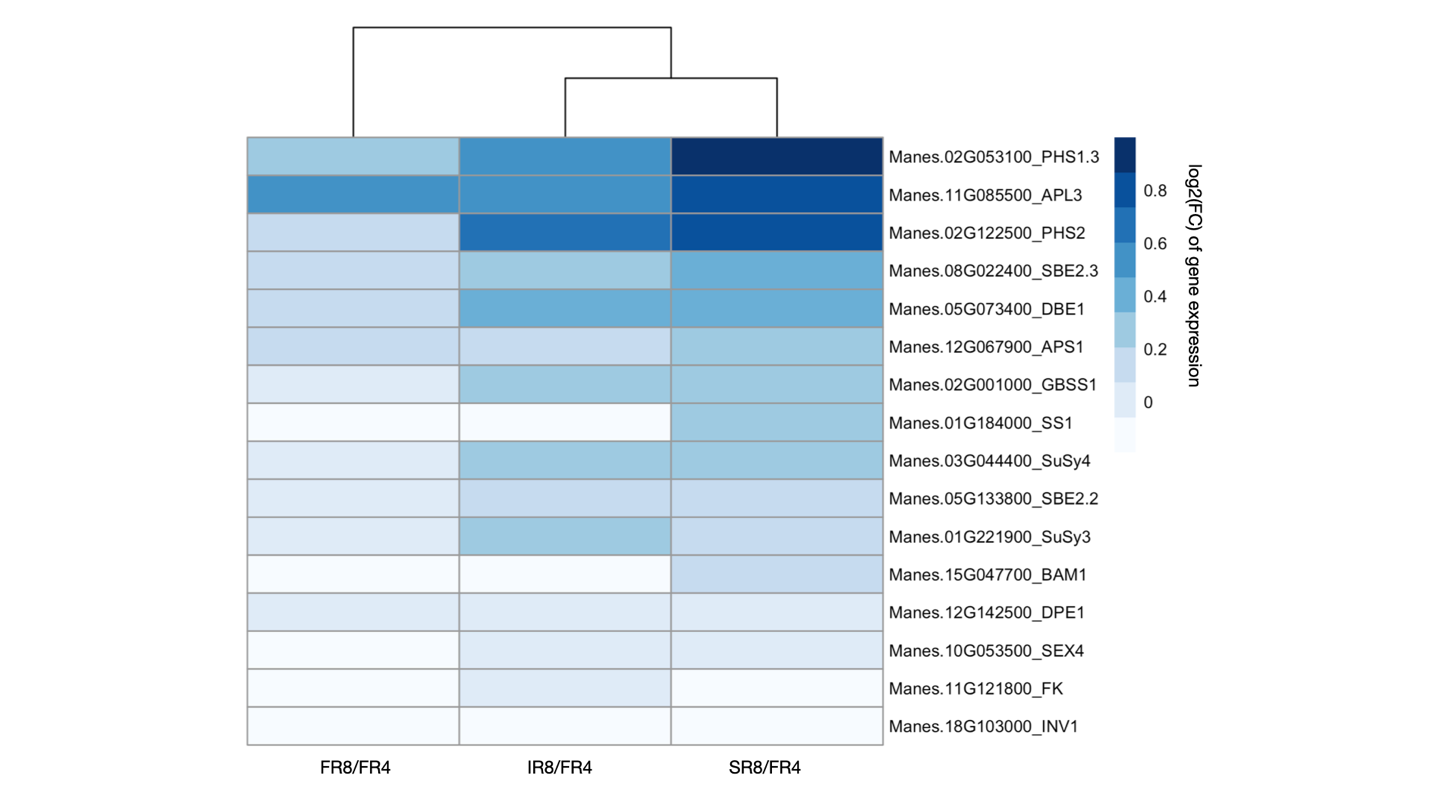
**

**Supplementary Figure S5.** Changes in expression of sucrose and starch-related genes during cassava SR development. The genes, namely *PHS1.3* (Glycosyl transferase, family 35), *APL3* (Glucose-1-phosphate adenylyltransferase family protein), *PHS2* (alpha-glucan phosphorylase 2), *SBE2.3* (starch branching enzyme 2.2), *DBE1* (debranching enzyme 1), *APS1* (ADP glucose pyrophosphorylase 1), *GBSS1* (UDP-Glycosyltransferase superfamily protein), *SS1* (Glycogen/starch synthases, ADP-glucose type), *SuSy4* (sucrose synthase 4), *SBE2.2* (starch branching enzyme 2.1), *SuSy3* (sucrose synthase 3), *BAM1* (beta-amylase 1), *DPE1* (disproportionating enzyme), *SEX4* (dual specificity protein phosphatase (DsPTP1) family protein), *INV1* (cytosolic invertase 2), and *FK* (pfkB-like carbohydrate kinase family protein), were proposed as key regulators in sucrose and starch metabolism by Ding et al. (2020) ^[3]^. The colors represent levels of gene expression, from low (white) to high (blue), obtained in this study. FR, IR, and SR denote fibrous, intermediate and storage roots, respectively, at four and eight weeks after planting, as indicated.


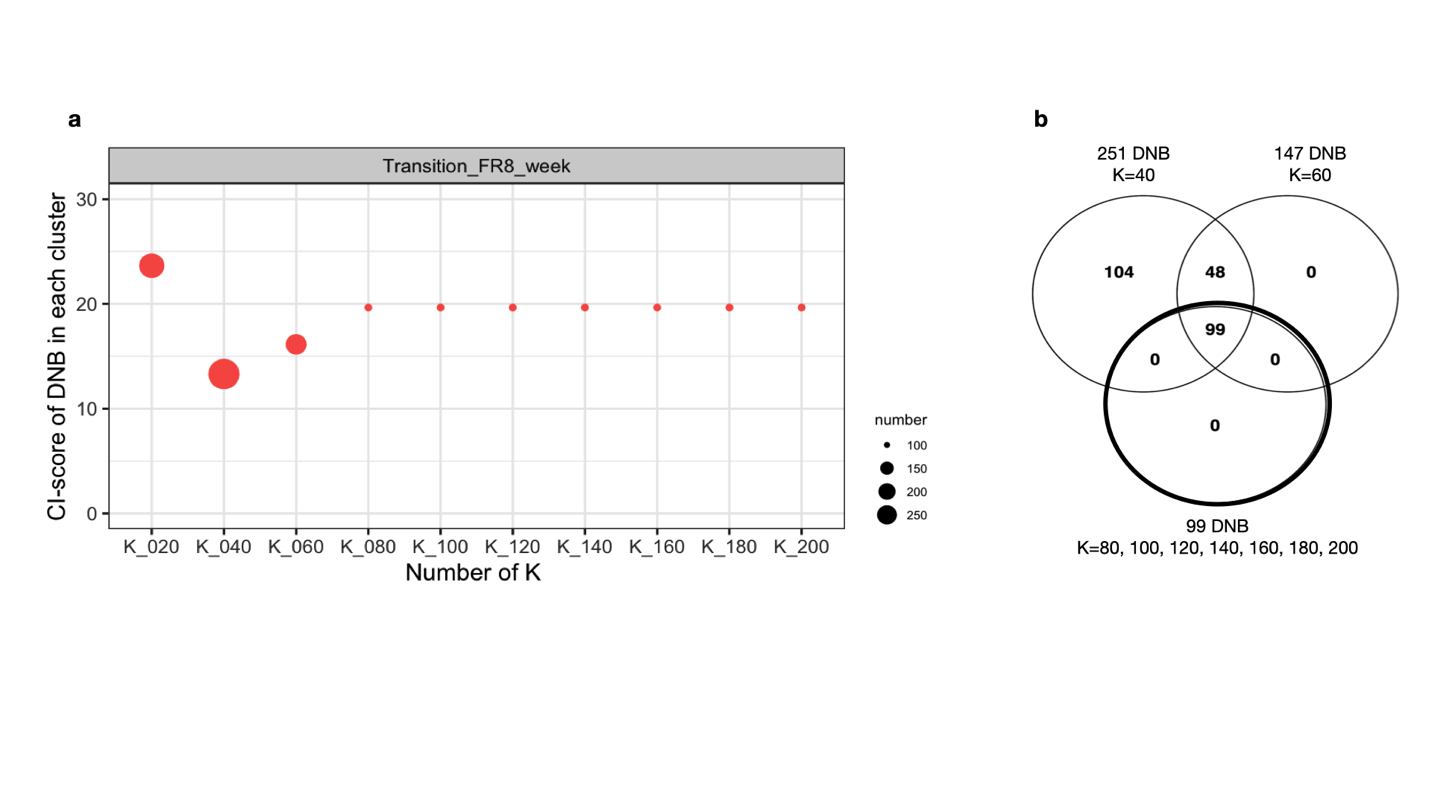


**Supplementary Figure S6. (a)** The CI score for each number of clusters (K 20 to 200). **(b)** The Venn-diagram of DNB genes in each number of clusters from 20 to 200.

**Reference**

1. Utsumi, Y. *et al.* Integrative omics approaches revealed a crosstalk among phytohormones during tuberous root development in cassava. *Plant Mol Biol* **109**, 249–269 (2020).

2. Rüscher, D. *et al.* Auxin signaling and vascular cambium formation enable storage metabolism in cassava tuberous roots. *J Exp Bot* **72**, 3688–3703 (2021).

3. Ding, Z. *et al.* Highly dynamic, coordinated, and stage-specific profiles are revealed by a multi-omics integrative analysis during tuberous root development in cassava. *J Exp Bot* **71**, 7003–7017 (2020).
